# β-Catenin Signaling Evokes Hair Follicle Senescence by Accelerating the Differentiation of Hair Follicle Mesenchymal Progenitors

**DOI:** 10.3389/fcell.2022.839519

**Published:** 2022-04-04

**Authors:** Jimin Han, Kaijun Lin, Huiqin Choo, Jia He, Xusheng Wang, Yaojiong Wu, Xiaodong Chen

**Affiliations:** ^1^ Department of Burn Surgery, The First People’s Hospital of Foshan, Foshan, China; ^2^ School of Life Sciences, Tsinghua University, Beijing, China; ^3^ The Shenzhen Key Laboratory of Health Sciences and Technology, Shenzhen, China, Graduate School at Shenzhen, Tsinghua University, Shenzhen, China; ^4^ School of Pharmaceutical Sciences, Sun Yat-sen University, Guangzhou, China; ^5^ Tsinghua-Berkeley Shenzhen Institute (TBSI), Tsinghua University, Shenzhen, China

**Keywords:** β-catenin, CD34, hair follicle dermal stem cells, dermal fibrosis, hair loss, aging

## Abstract

**Rationale:** β-catenin signaling controls multiple fibroblast subsets, with its overactivity promoting the differentiation of hair follicle dermal stem cells (hfDSCs) and the hyperactivation of interfollicular fibroblasts. Understanding the concept of hfDSC activation and modulation offers hope towards the therapeutic armamentarium in dermatology and related comorbidities, as well as their potential applications in gerontology (the study of physiological aging). Having a comprehensive understanding in this stochastic process could also further yield important, novel insights into the molecular basis of skin aging to improve lifespan and preventing aging-related diseases.

**Methods:** A new CD34CrePGR mouse line was generated. Through fate-tracing models and a series of β-catenin genetic experiments, our study depicts how the wound environment increases phosphorylated β-catenin in hfDSCs and facilitates their differentiation into dermal papilla (DP) and dermal sheath (DS). In mice carrying hfDSC-specific activated allele of β-catenin, hfDSCs accelerated their differentiation into DP cells.

**Results:** Notably, with β-catenin stabilization in CD34-expressing cells and potential activation of canonical Wnt signaling, the mutant mice showed a brief increase of hair density in the short term, but over time leads to a senescence phenotype developing premature canities and thinning [hair follicle (HF) miniaturization].

**Conclusion:** β-catenin signaling drove HF senescence by accelerating differentiation of CD34^+^ hfDSCs, resulting in phenotypes attributable to the differentiation of the hfDSCs into DP cells and the loss of their stem cell potential. Therefore, our study reveals that the regulation of β-catenin signaling in hfDSCs may potentially become an important subject for future exploration in development of clinically effective therapies for hair loss treatment and an excellent model for revealing new therapeutic approaches to reverse aging or retarding the development of alopecia.

## 1 Introduction

Aging is a physiological process in which the structural and regulatory mechanisms of tissues and organs gradually leads to intrinsic changes also known as senescence, which is mediated by numerous biological and genetic pathways (Shin et al., 2020). The functional and growth capacity of hair follicles (HFs) are highly influenced and modulated by age, and hair loss or alopecia is present in both accelerated aging mouse models and chronic aging in humans due to hair cycling defects (Harrison and Archer, 1988; Courtois et al., 1995; Geyfman and Andersen, 2010). During the physiological aging process, the mesenchymal niche of HFs, including dermal papilla (DP) and dermal sheath (DS), is gradually impaired and the epidermal component of HFs is unable to regenerate hair due to its inability to receive signals from its dermal components (Elliott et al., 1999; Shin et al., 2020). DP is a major dermal compartment in the HFs, which is the key signal transduction center for impeding hair shaft growth, HF circulation and pigmentation (McElwee et al., 2003; Enshell-Seijffers et al., 2010; Chi et al., 2013). When the DP cell density falls below a critical threshold, the epidermal cells of HFs are unable to generate new hair shaft (Chi et al., 2013).

Stem cell (SC) exhaustion has been proposed to be one of the crucial driving factors of age-associated tissue degeneration (Liu et al., 2013; Lopez-Otin et al., 2013). Previous findings have shown that at the start of each new hair cycle, there is a pool of long-lived, self-renewing, bipotent hair follicle dermal stem cells (hfDSCs) progeny commit to either DS or DP fates (Biernaskie et al., 2009; Rahmani et al., 2014; Shin et al., 2020). For example, through advancement of age, hfDSCs self-renewal activity gradually declines, then undergo terminal differentiation and eventually exhaustion, eventually leads to a series of pathological manifestations (Shin et al., 2020). The impairment of hfDSCs is known to damage the supplement of DP cells and leads to progressive HF senescence or are subject to apoptosis (Shin et al., 2020). Senescent cells secrete a large number of inflammatory factors and present with the senescence-associated secretory phenotype (SASP), which has detrimental effects on the surrounding environment and excessive accumulation of senescent cells over time inevitably affects tissue regeneration capacity and homeostasis (Elliott et al., 1999; Shin et al., 2020). It has also been reported that age-related accumulation of senescent cells has been observed in the dermis and is regarded as a driver of age-dependent pathologies (McElwee et al., 2003; Enshell-Seijffers et al., 2010; Chi et al., 2013).

Wnt family members are known to participate a variety of physiological and pathological processes, controlling SC differentiation, tissue morphogenesis during homeostasis and immune cell maintenance (including HF development), and aging (Ito et al., 2007; Enshell-Seijffers et al., 2010; Tsai et al., 2014; Kaur et al., 2016; Tao et al., 2019). β-catenin signaling is essential for dermal homeostasis and the progression of dermal fibrosis (Mastrogiannaki et al., 2016; Mok et al., 2019). Conditional activation of β-catenin in hfDSCs leads to their cellular fate decision, thereby differentiating into abnormal dermal fibroblasts or acquiring a tendency to transition to DP cells (Tao et al., 2019). However, whether β-catenin is the molecular signal that regulates the differentiation of hfDSCs as of today, still remains incompletely understood.

CD34 is a glycophosphoprotein expressed on the surface of most hematopoietic progenitor cells. After mobilization, CD34^+^ cells accounted for 1%∼5% of peripheral blood mononuclear cells. CD34 is widely used to obtain SCs for autologous bone marrow transplantation (Ito et al., 2007; Enshell-Seijffers et al., 2010; Tsai et al., 2014; Kaur et al., 2016; Tao et al., 2019). CD34 is also found in several non-hematopoietic tissues, including skeletal muscle, gastrointestinal tract, and endothelial cells (Ito et al., 2007; Enshell-Seijffers et al., 2010; Tsai et al., 2014; Kaur et al., 2016; Tao et al., 2019). In addition, a large body of data shows that CD34, is uniquely expressed on murine HF bulge keratinocytes, which is also expressed in both epithelial and mesenchymal components of anagen human HFs (Ito et al., 2007; Enshell-Seijffers et al., 2010; Tsai et al., 2014; Kaur et al., 2016; Tao et al., 2019). Although CD34 was found to be expressed in slightly more differentiated outer root sheath cells below the bulge in the human HF, however, research has also indicated that the role of CD34 is very important for hair matrix or HFSCs as results have conveyed that CD34^+^ cells were able to regenerate more HFs than CD34^−^ cells. Even though other research has proven that CD34 is not specific marker for human bulge cells, however this marker was found to facilitate isolation of live epithelial cells with stem and progenitor cell characteristics. Further intensive research on CD34 has also concluded that not all human HFSCs compartments may exist in the bulge region (Ito et al., 2007; Enshell-Seijffers et al., 2010; Tsai et al., 2014; Kaur et al., 2016; Tao et al., 2019).

Using CD34 as a genetic marker, we developed a CD34-promoter-driven CrePGR mouse model to offer an alternative insight on the optimization of HF-derived SC sources for clinical applications. Further investigations of SC niches will contribute to the development of hair regenerative therapy as a prominent class of organ replacement model for pre-clinical therapeutic evaluation and regenerative therapy in the future through permit cell fate tracing of hfDSCs. CD34^+^ cells are also found to be a specific marker for adipose-derived mesenchymal stem cells (ADMSCs) in hair morphogenesis (He et al., 2013).

During the hair cycle, hfDSCs which drives the cyclic renewal of the dermal sheath (DS), are heterogeneous and are housed during the growth phase within the most proximal part of the DS (Aamar et al., 2021). Therefore, by using a lineage-specific tracing approach, we examined the effects and consequences of β-catenin stabilization in CD34^+^ hfDSCs. During lineage tracing, CD34^+^ hfDSCs differentiated into DP and DS cells at each anagen. The results from other studies have also demonstrated that only CD34^+^ cells were able to participate in HF morphogenesis by contributing to the DS formation (He et al., 2013). Therefore in this study, we used CD34CrePGR: mTmG:Ctnnb1^tm1Mmt/+^ (referred to as CD34:mTmG:β-cat^act^) mice as a HF reconstitution model to stabilize β-catenin in CD34^+^ cells, and found that CD34^+^ hfDSCs with stabilized β-catenin accelerated their differentiation into DP and DS. Data has also shown that DP and DS cells are confined to the mesenchymal compartments of the HF during homeostasis (Aamar et al., 2021). Furthermore, our studies found that β-catenin activation in CD34^+^ cells led to senescence and depletion of hfDSCs. However, in contrast, β-catenin knockout significantly inhibited the differentiation of CD34^+^ hfDSCs into DP. After an injury, β-catenin is found activated and would accelerate the differentiation of hfDSCs. Stabilization of β-catenin in CD34^+^ cells led to senescence of mesenchymal cells and the formation of progressive alopecia.

Indeed from this, we further denoted the unique biological properties of CD34^+^ hfDSCs, making it a highly promising potential molecular target for regulating HF senescence with clinical applications towards cell-based approaches for alopecia treatment (Shin et al., 2020).

## 2 Results

### 2.1 CD34 is a Putative Marker of hfDSCs

**FIGURE 1:**
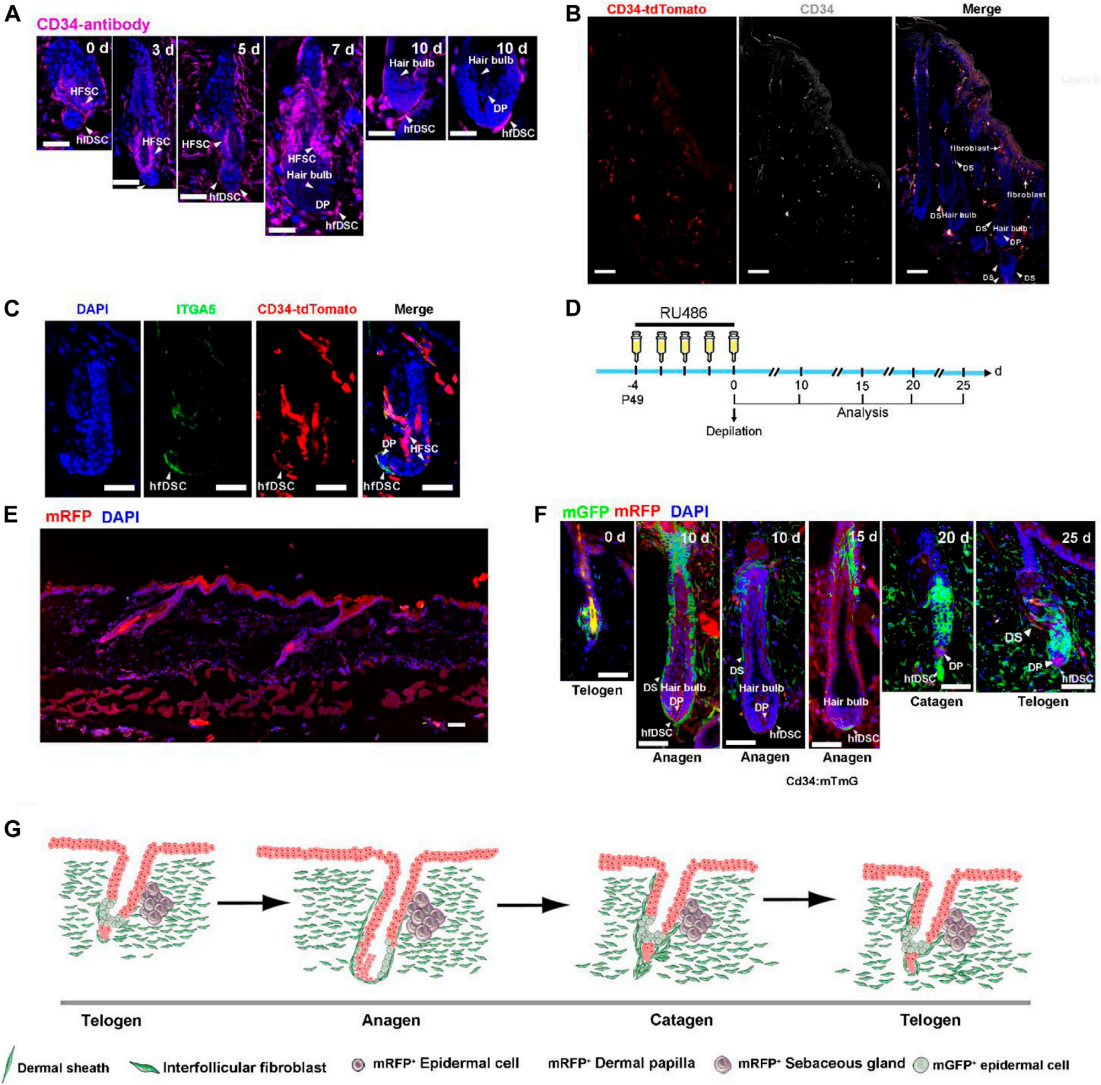
CD34 is expressed in hfDSCs adjacent to DP and dermal fibroblasts. **(A)** Immunostaining analysis for CD34 (purple) in skin tissues of C57BL/6 mice 0, 3, 5, 7 and 10 days after depilation. Arrowheads point to DS cells that express CD34. **(B)** Confocal microscope images of skin sections from CD34-tdTomato (red) mice immunolabeled with CD34 (white). Note the colocalization of tdTomato with CD34 in DS and fibroblasts. **(C)** Immunofluorescence analysis of a telogen HF from CD34-tdTomato (red) mice immunolabeled with ITGA5 (green). **(D)** Experimental scheme for RU486 injection and analysis of HFs at different stages of the depilation-induced hair cycle in CD34:mTmG mice. **(E)** Images of skin sections harvested from CD34:mTmG without RU486 injection. **(F)** Representative images showing the fate of CD34-lineage cells (green) at each stage of the first depilation-induced hair cycle in 7-week-old CD34:mTmG mice. **(G)** The schematic illustrating the tracing of CD34-lineage cells (green) during the first depilation-induced hair cycle. Scale bars, 50 μm.

CD34 is identified as a typical mesenchymal cell marker, which is the hallmark of interfollicular fibroblasts in the skin (Rinkevich et al., 2015; Salzer et al., 2018; Shook et al., 2018). Although CD34 is known to be a glycoprotein labelling hematopoietic progenitors, it is also used as a marker of epithelial SCs in the HF (Trempus et al., 2003). This molecule has also been reported to be present in the mesenchymal compartment (Lin et al., 2015). Previous evidence has linked CD34 to putative dermal stem cells located in the murine whiskers which demonstrated its capacity to differentiate into several mesenchymal lineages (Hoogduijn et al., 2006). Motivated by this, our initial validation was focused on the speculation that CD34 could be an important marker of hfDSCs. We first analyzed the CD34 expression of the skin during hair cycle. C57BL/6 mice were depilated to induce anagen, and skin samples were collected 0, 3, 5, 7 and 10 days postdepilation, and then stained with CD34 (Figure 1A). Although some studies suggested that CD34 expression was rarely detected in Sox2-positive hfDSCs and DP cells (Biernaskie et al., 2009), specific CD34 expression was detected within hfDSCs located at the periphery of telogen DP (Figure 1A). During anagen, CD34 expression was found in the dermal cup (DC), defined as the pool of hfDSCs (Figure 1A). Importantly, CD34 expression was not observed in DP cells throughout the hair cycle (Figure 1A), which was consistent with previous studies on the endogenous CD34 mRNA expression in the DP (Joost et al., 2020).

To further assess the characteristics of CD34^+^ hfDSCs, we made an attempt to recreate the CD34-CrePGR mouse line that expresses CrePGR under the direct control of the CD34 promoter (Jiang et al., 2021). With much success, we managed to generate a CD34-CrePR1-P2A-tdTomato mouse model, in which tdTomato fluorescent protein is selectively expressed in CD34^+^ cells (Figures 1B,C). Indeed, immunostaining with CD34 demonstrated the effectiveness of tdTomato labeling. As previously reported (Blanpain et al., 2004; Rinkevich et al., 2015; Salzer et al., 2018; Shook et al., 2018), immunostaining also showed a clear indication that tdTomato fluorescence was present in the bulge and dermal fibroblasts, and largely overlapped with areas stained with CD34 (Figure 1B). Staining of CD34-CrePR1-P2A-tdTomato skin sections with ITGA5 also revealed that tdTomato^+^ cells existed within the DS (Figure 1C).

### 2.2 The Tracking of CD34^+^ Cells Further Evince the Expression Pattern of CD34

To further verify the effectiveness of our transgenic mice model, lineage tracing was conducted to verify the endogenous promoter activity of the CD34 gene. We crossed CD34-CrePR1-P2A-tdTomato mice with Rosa26-mTmG reporter mice to generate CD34CrePGR:Rosa26-mTmG mice (referred to as CD34:mTmG), which enable RU486-inducible, irreversible membrane GFP (mGFP) expression in CD34^+^ cells. Notably, without RU486 treatment, mGFP^+^ cells were not detected (Figure 1E). The CD34:mTmG mice received RU486 injections at P49 for five consecutive days, and then depilated to further induce a new hair cycle (experimental schematic, see Figure 1D). During telogen, 5 days postdepilation, mGFP expression was detected within hfDSCs, as indicated by their colocalization with ITGA5 (Supplementary Figure S1A). During anagen, mGFP-labeled areas expressed ITGA5, confirming their extensive expression in DS (Supplementary Figure S1B). With the transition to catagen phase, the elongated mGFP-marked hfDSC pool showed high labeling efficiency (20–25 days) (Figure 1F). The degenerative hfDSCs also showed faint colocalization with ITGA5 (Supplementary Figure S1C). Collectively, these data showed a clear indication of the fidelity of our transgenic model in mimicking the endogenous CD34 expression.

### 2.3 CD34^+^ hfDSCs Replenish DP and DS Over Consecutive Hair Cycles

**FIGURE 2:**
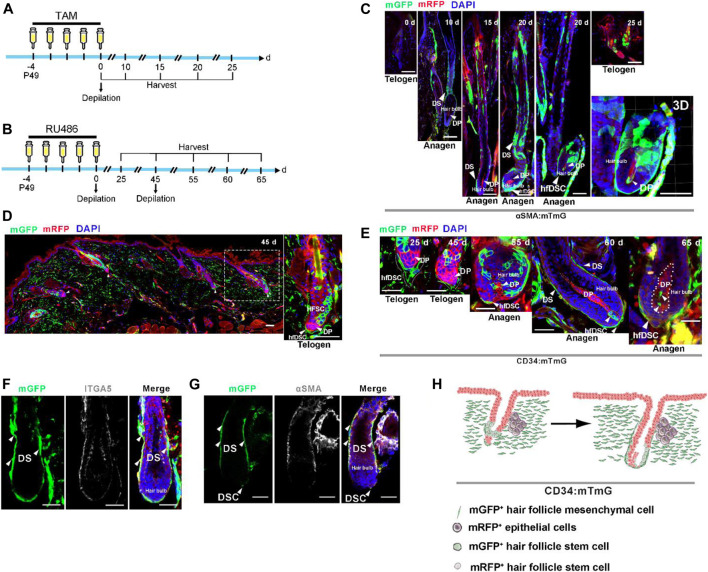
CD34^+^ hfDSC progeny repopulate DS and DP cells during an ongoing hair cycle. **(A)** Outline of lineage-tracing strategy in αSMA:mTmG mice. **(B)** Experimental design showing fate mapping assessment of CD34^+^ hfDSCs labeled at P49-53 and harvested at different stages of the hair cycle. **(C)** Typical images of HFs in αSMA:mTmG mice at different stages. Confocal reconstructions of an anagen HF showed that αSMA^+^ hfDSCs supplemented DP cells (right-most panel). **(D)** Representative telogen HFs 45 days post first depilation showing the distribution of mGFP fluorescent protein in hfDSCs (left panel). A higher-magnification view of the left panel (right panel). **(E)** Fate of CD34-lineage cells (green) at different stages of the hair cycle. Dotted lines: approximate location of DP. **(F)** ITGA5 immunostaining of an anagen HF from CD34:mTmG mice. **(G)** An anagen HF from CD34:mTmG mice immunostained for αSMA. αSMA, alpha smooth muscle actin. **(H)** Schematic summary of the fate of CD34-lineage cells (green) during the second depilation-induced hair cycle. Scale bars, 50 μm.

Considering the ability of αSMA^+^ hfDSCs to reconstitute HF mesenchyme (Rahmani et al., 2014; Hagner et al., 2020; Shin et al., 2020), we questioned whether CD34^+^ hfDSCs contributed to the maintenance of DP and DS. We first utilized previously reported transgenic mice containing αSMACreERT2 (expressing tamoxifen-inducible Cre) (Wu et al., 2007) to verifying the capacity of hfDSCs to differentiate into DP by mating αSMACreERT2 to Rosa26-mTmG reporter mice (referred to as αSMA:mTmG). Upon tamoxifen application, lineage tracing of αSMA^+^ hfDSCs was performed after depilation (experimental schematic, see Figure 2A). Indeed, hfDSC progeny was clearly observed within DP when HFs entered anagen (Figure 2C).

We also conducted lineage tracing of CD34^+^ hfDSCs in the second depilation-induced hair cycle. After initial depilation, HFs once again entered into telogen phase, the skin tissue showed clearly visible mGFP-labeled hfDSCs situated exclusively around DP with a high labeling efficiency (Figure 2D). These labeled cells colocalized with ITGA5 (Supplementary Figure S1D). Then, a second depilation was performed to induce another hair cycle (experimental schematic, see Figure 2B). Ten days post second depilation, two to three mGFP-labeled cells were located in DC (Figure 2E). As reported (Rahmani et al., 2014; Hagner et al., 2020; Shin et al., 2020), an expansion of CD34^+^ hfDSC progeny could be occasionally detected within anagen DP 15–20 days after the second depilation (Figure 2E). Staining with ITGA5 showed that the mGFP-expressing repopulated areas contained DS (Figures 2F,G; Supplementary Figure S1E). The paradigms we have unearthed suggest that CD34^+^ hfDSCs exhibited obvious bidirectional differentiation (DP and DS) (Figure 2H).

### 2.4 Wound Healing Process Accelerates the Recruitment of CD34^+^ hfDSC Progeny to the DP and DS Adjacent to Wounds

Wound healing holds strong resemblance to HF development as it requires a highly coordinated interplay between cell migration, proliferation and growth (Shin et al., 2020). Studies have shown that wound-induced hair anagen (WIHA) usually occurs in the skin close to the cutaneous wounds (Argyris, 1956; Wang et al., 2017). Based on this phenomenon, we hypothesized that wound injury stimulates the differentiation of hfDSCs into DP cells, thereby replenishing DP cells and inducing hair growth. Thus, biases the fate choice of hfDSC progeny. To label hfDSCs as completely as possible, CD34:mTmG mice received RU486 injections and were subjected to a full depilation-induced hair cycle. When HFs progressed to the next telogen, we introduced a wound with a diameter of 1.5 cm into the dorsal skin. Twenty days postwounding, we removed the skin adjacent to the wounds (experimental schematic, see Supplementary Figure S2A). Intriguingly, DS in wound proximity were mostly mGFP-positive (Supplementary Figure S2B), and such high labeling efficiency was not observed in DS during homeostasis. These findings provided compelling evidence indicating that a wound environment promoted differentiation of CD34^+^ hfDSCs into DS cells. Under normal circumstances, hfDSC progeny fill only the DP below Auber’s line (Shin et al., 2020). Significantly, in the HFs situated close to wounds, CD34-derived descendants were no longer confined to the area below Auber’s line but extended into the DP above Auber’s line (Supplementary Figure S2C), further confirming that the environment can regulate the hair regeneration process. Notably, immunostaining showed that mGFP colocalized with ITGA5 in DP at the wound edge, indicating that DP cells originating from CD34^+^ hfDSCs still retained their original phenotype (Supplementary Figure S2C). We concluded that the wound environment enhanced the migration of hfDSC progeny to replenish DP (Supplementary Figure S2D), and the swift change in cell fate decision determined that CD34^+^ hfDSCs did not lose their original expression.

β-catenin signaling pathway plays an important role in cell fate determination, proliferation and differentiation (Shin et al., 2020). Stabilization of β-catenin in epidermis induces ectopic HF regeneration, and even pathogenesis of neoplasm. It also was recently reported that excessive β-catenin promotes the upregulation of DP-related signature genes in hfDSCs (Tao et al., 2019). Therefore, we hypothesized that the damaged environment might increase β-catenin signaling in hfDSCs. To test this possibility, we examined whether phosphorylated β-catenin (p-β-catenin) expression was altered in the wounded environment. Interestingly, in HFs adjacent to wounds, cutaneous injury elicited an elevation of p-β-catenin expression in hfDSCs located within DC (Supplementary Figure S2E-F), indicating active β-catenin signaling in hfDSCs. Taken together, these data indicated that the traumatic environment promoted the activation of β-catenin in hfDSCs, and therefore, β-catenin signaling in hfDSCs might play an essential role in hfDSC differentiation. This observation, as well as the effects of β-catenin signaling on hfDSCs acquiring the propensity to transition to DP (Tao et al., 2019), prompted us to further investigate its activity in hfDSCs which may unlock novel hair-growth stimulating therapeutics.

### 2.5 β-Catenin Stabilization in CD34^+^ Cells Results in Advanced Anagen Initiation and hfDSC Terminal Differentiation

**FIGURE 3:**
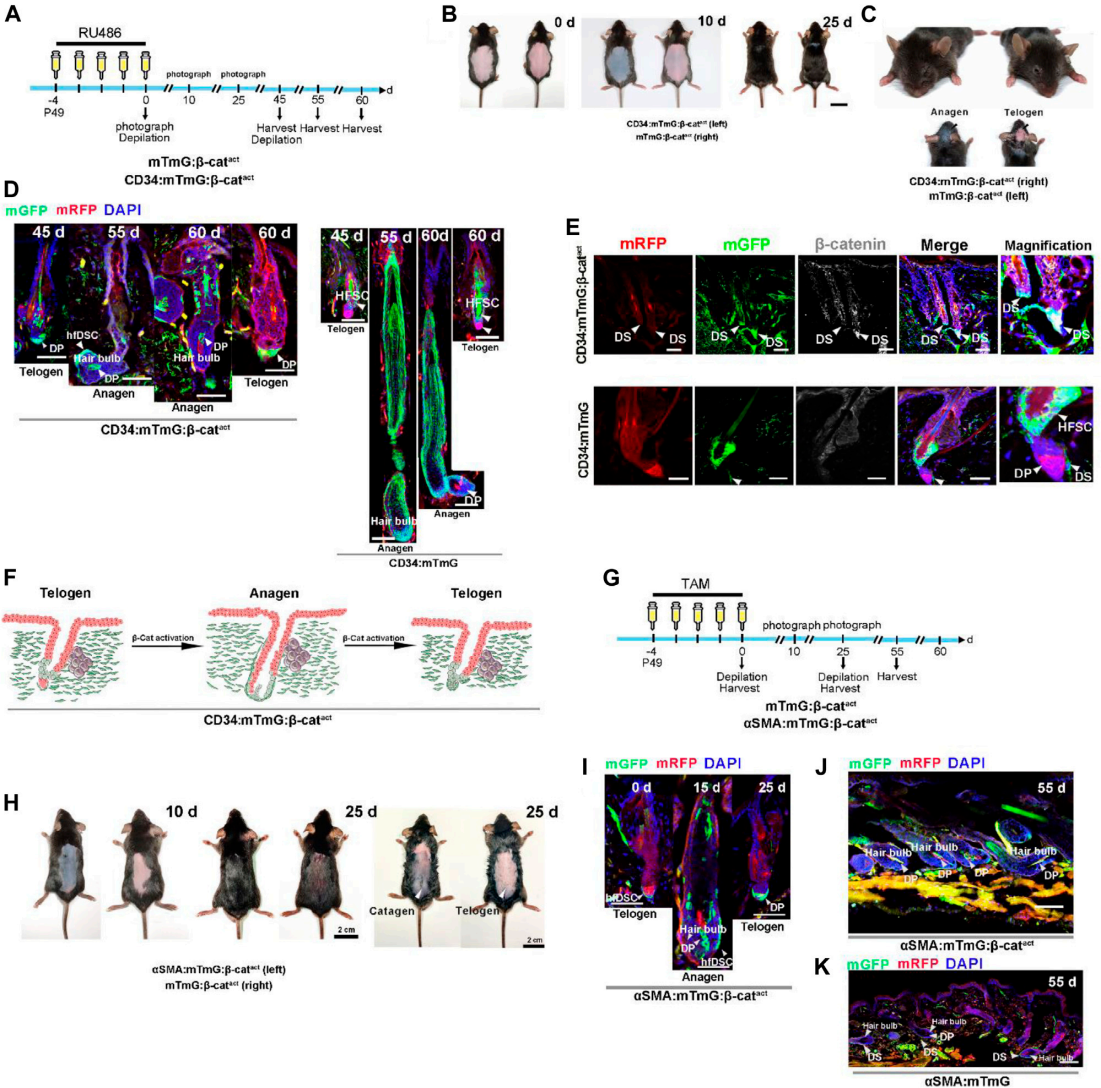
Activation of β-catenin signaling results in accelerated differentiation of hfDSCs into DP cells. **(A)** Experimental timeline of RU486 injections and tissue collections in CD34:mTmG:β-cat^act^ mice and control mTmG:β-cat^act^ littermates. **(B)** Representative pictures of dorsal skin from CD34:mTmG:β-cat^act^ mice and control mTmG:β-cat^act^ littermates. **(C)** Photograph features of the heads of CD34:mTmG:β-cat^act^ mice and control mTmG:β-cat^act^ littermates. Note that HFs from the head skin of CD34:mTmG:β-cat^act^ mice were at anagen. **(D)** Representative images showing HFs at different stages of the second depilation-induced hair cycle. Note that mGFP-labeled cells occupied almost the entire DP in CD34:mTmG:β-cat^act^ mice. **(E)** Expression of β-catenin (white) was observed by immunofluorescence in the HFs of control CD34:mTmG (lower panel) and CD34:mTmG:β-cat^act^ (upper panel) mice. Note nuclear β-catenin expression in hfDSCs of CD34:mTmG:β-cat^act^ mice. **(F)** Schematic diagram of the supplementation of DP by CD34^+^ hfDSC progeny (green) upon β-catenin activation. **(G)** Experimental timeline of tamoxifen injections and tissue collections in αSMA:mTmG:β-cat^act^ mice and control mTmG:β-cat^act^ littermates. **(H)** Hair coats of pairs of αSMA:mTmG:β-cat^act^ (left panel) and mTmG:β-cat^act^ (right panel) mice were photographed 10 and 25 days after tamoxifen injections. Shaved (middle panel) and depilated (right panel) dorsal skin showed that HFs were still at catagen on the 25th day. **(I)** Representative images showing HFs in αSMACreER:mTmG mice at different stages of the hair cycle. **(J,K)** Representative pictures of dorsal skin from αSMA:mTmG and αSMA:mTmG:β-cat^act^ mice on the 55th day. Scale bars, 2 cm **(B,C,H)**, 50 μm **(D,E,I,J,K)**.

Based on the above results, we postulated that β-catenin signaling might control hfDSC differentiation. To assess the function of β-catenin in CD34^+^ hfDSC differentiation, we bred homozygous Ctnnb1^tm1Mmt^ mice into CD34:mTmG mice to generate mice carrying a conditional activated allele of β-catenin (CD34:mTmG:β-cat^act^). Both CD34:mTmG:β-cat^act^ mice and control mTmG:Ctnnb1^tm1Mmt/+^ (referred to as mTmG:β-cat^act^) littermates received RU486 injection for five consecutive days followed by depilation (experimental schematic, see Figure 3A). Analysis of dorsal skin revealed that β-catenin activation in CD34^+^ cells triggered precocious anagen entry 10 days postdepilation, while HFs of control mice were still at telogen-anagen transition (Figure 3B; Supplementary Figures S3A,B). Twenty days postdepilation, excess β-catenin signaling led to a growth phase in both the dorsal skin and the head skin that was not depilated, and hair density was much higher than that of the control mice (Figures 3B,C; Supplementary Figures S3C–F). These data demonstrated that β-catenin stabilization in CD34^+^ cells not only intensified depilation-induced anagen entry, but also triggered active spontaneous hair anagen to head telogen HFs.

Digging deeper, we next performed lineage tracing in CD34:mTmG:β-cat^act^ mice during consecutive regenerative cycles (experimental schematic, see Figure 3A). Confirmation of nuclear β-catenin expression in hfDSCs was shown in Figure 3E and Supplementary Figures S4A.B. During homeostasis, hfDSC progeny recruited into the DP will reintegrate into the hfDSC niche during catagen (Rahmani et al., 2014). This revealed the possibility that differentiated hfDSC progeny exhibit plasticity that enables them to reconvert to SC states. Surprisingly, in CD34:mTmG:β-cat^act^ mice, on day 0 after the second depilation, mGFP-labeled cells occupied almost the entire resting DP (Figure 3D), suggesting that hfDSC progeny could not repopulate the SC pool. When HFs entered anagen, both supplementary and definitive DP cells were almost completely labeled by mGFP (Figure 3D; Supplementary Figure S5A). At the end of the second depilation-induced hair cycle, mGFP-labeled cells also persisted in the telogen DP (Figure 3D). The above results indicated that hyperactive β-catenin signaling expedite CD34^+^ hfDSC differentiation, but this was accompanied by a rapid loss of hfDSC plasticity.

### 2.6 Selective β-Catenin Activation in αSMA^+^ hfDSCs Resulted in Acceleration of Anagen Initiation and hfDSC Terminal Differentiation

We next examined whether a similar phenomenon existed when β-catenin is selectively activated in hfDSCs by performing similar experiments in αSMA^+^ cells to support our hypothesis. Homozygous Ctnnb1^tm1Mmt^ lines were crossed with αSMA:mTmG mice to obtain αSMACreER:Rosa26-mTmG:Ctnnb1^tm1Mmt/+^ (referred to as αSMA:mTmG:β-cat^act^) mouse lines. Daily tamoxifen administration was initiated when αSMA:mTmG:β-cat^act^ mice and control mTmG:β-cat^act^ littermates were 7-week-old, and depilated back skin were analyzed 10 and 25 days following injections (experimental schematic, see Figure 3G). On the 10th day after depilation, HFs in αSMA:mTmG:β-cat^act^ mice were in the anagen phase, compared to the telogen phase in the control mTmG:β-cat^act^ littermates (Figure 3H; Supplementary Figures S3G–H). At day 25 post depilation, shaved back skin of αSMA:mTmG:β-cat^act^ mice showed dense and glossy hair, whereas control mice displayed relatively sparse hair (Figure 3H; Supplementary Figures S3I,J). Depilated back skin of αSMA:mTmG:β-cat^act^ mice also showed an apparent catagen phase, whereas that of control mice exhibited an obvious telogen phase (Figure 3H).

We then conducted lineage tracing experiments in αSMA:mTmG:β-cat^act^ mice (experimental schematic, see Figure 3G). After tamoxifen injections, hfDSCs in telogen HFs were clearly marked (Figure 3I; Supplementary Figure S6A). When the HFs entered anagen, hfDSCs generated progeny to replenish DP cells, some of which were located in the definitive DP (Figure 3I; Supplementary Figures S6B–D). Importantly, mGFP^+^ hfDSC progeny persisted in the telogen DP (Figure 3I; Supplementary Figure S6E), as observed in CD34:mTmG:β-cat^act^ mice. At the second depilation-induced anagen, αSMA^+^ hfDSC progeny populated both supplementary and definitive DP cells (Figure 3J; Supplementary Figures S5C.D, S6F). Taken together, we concluded that constitutive β-catenin stabilization drove the terminal differentiation of hfDSCs (Supplementary Figures S6D,G).

### 2.7 Activation of β-Catenin Signalling Promotes Dermal Fibrosis

During lineage tracing of the CD34:mTmG:β-cat^act^ mice (experimental schematic, see Figure 3A), the dorsal skin 1 day postinjection displayed prominent fibrotic areas that had largely replaced the adipocyte layer (Supplementary Figures S7A,B). With time, activated CD34^+^ fibroblasts transformed into abnormal fibroblasts and continued to expand in the dermis (Supplementary Figures S7C,D). By 20 days postinjection, both the upper and lower dermis demonstrated significant fibrosis, suggesting that excess activity of β-catenin promoted hyperactivation of CD34^+^ fibroblasts in both the upper and lower dermis (Supplementary Figures S7E,F).

In the current study, we also examined the skin slices of αSMA:mTmG:β-cat^act^ mice. The mutant transgenic mice developed a prominent degree of fibrosis in the lower dermis over time (Figure 3J; Supplementary Figures S6A–C, S7C,D), which is consistent with prior observation (Tao et al., 2019), suggesting that β-catenin is essential for the conversion of hfDSCs to the more terminally differentiated interfollicular fibroblasts. This further supports the idea that β-catenin signaling has a regulatory role in the differentiation of hfDSCs.

### 2.8 Ectopic Activation of β-Catenin Leads to hfDSC Depletion and HF Mesenchymal Senescence

**FIGURE 4:**
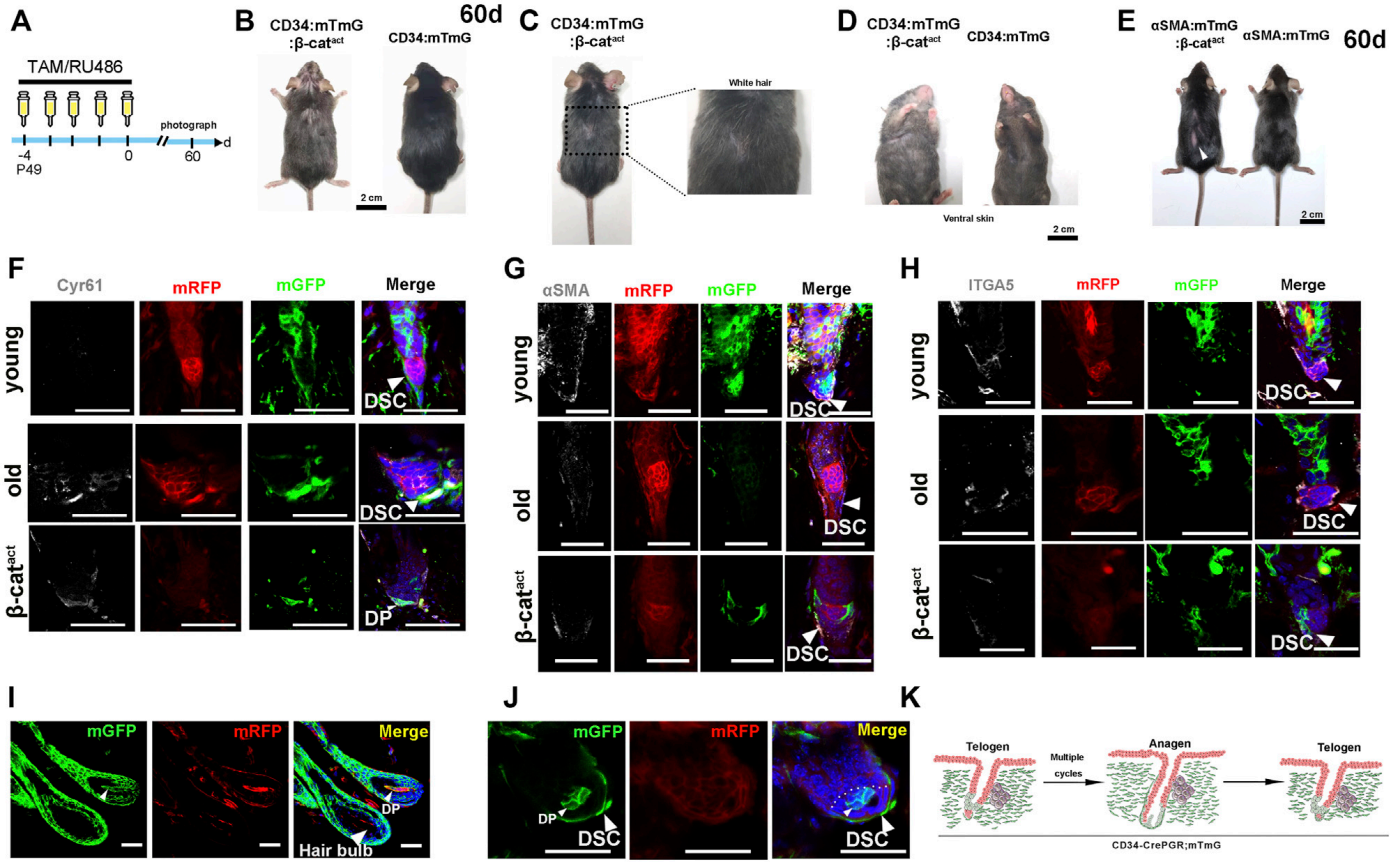
Activation of β-catenin leads to HF mesenchymal senescence and reduction of hfDSC progenitor pool. **(A)** Schematic depicting the timeline of RU486/tamoxifen injections and analysis. **(B)** Hair coat of CD34:mTmG:β-cat^act^ (left panel) and mTmG:β-cat^act^ (right panel) mice photographed at 60 days after RU486 injections. **(C)** CD34:mTmG:β-cat^act^ mice exhibited significantly grayer hair coats. **(D)** Ventral view of CD34:mTmG:β-cat^act^ mice (left panel) and control mTmG:β-cat^act^ littermates (right panel). **(E)** Hair coats of pairs of αSMA:mTmG:β-cat^act^ (left panel) and mTmG:β-cat^act^ (right panel) mice were photographed 60 days after tamoxifen injections. **(F,G)** Immunostaining for Cyr61 **(F)**, αSMA **(G)** and ITGA5 **(H)** in skin tissues from young (upper panel) and old CD34:mTmG mice (middle panel), and CD34:mTmG:β-cat^act^ mice (lower panel). **(I)** HFs of 18-month-old CD34:mTmG mice illustrating DP cells above Auber’s line replenished by mGFP-labeled cells. **(J)** Telogen HFs of 18-month-old CD34:mTmG mice showed a significant increase in lineage-labeled mGFP^+^ cells within DP. **(K)** Schematic illustration of the CD34 lineage tracing (green) in the HF of CD34:mTmG mice after multiple cycles. Scale bars, 2 cm **(B–E)**; 50 μm **(F–K)**.

We tracked long-term changes in CD34:mTmG:β-cat^act^ and αSMA:mTmG:β-cat^act^ mice (experimental schematic, see Figure 4A). Strikingly, mice from both genotypes exhibited premature hair loss 60 days after depilation (Figures 4B,E). In addition, extensive gray hair (canities) appeared on the dorsal and ventral skin of CD34:mTmG:β-cat^act^ mice (Figures 4C,D), indicating a severe hair senescence-associated phenotype. This discrepancy of these two genotypes of mice may be due to β-catenin signaling influencing CD34^+^ HF SCs and melanocyte stem SCs (Rabbani et al., 2011; Joshi et al., 2019). In light of these results, we hypothesized that the terminal differentiation of hfDSCs led to HF senescence.

To test this hypothesis, we first performed long-term tracing of CD34^+^ hfDSCs. We administered RU486 into CD34:mTmG mice aged 7 weeks and allowed them to go through hair cycles naturally until they were 1 year old when their dorsal skin was depilated. After two full depilation-induced hair cycles, we performed another depilation. Upon entry into the anagen phase of the third depilation-induced hair cycle, HFs were analyzed. As expected (Shin et al., 2020), a small portion of DP above Auber’s line was occupied by mGFP-labeled cells (Figure 4I). Additionally, when HFs entered telogen, a significant fraction of mGFP-labeled cells remained in the DP (Figure 4J). This indicated that hfDSC progeny that had left their niche could not withdraw from the DP to reconstruct the hfDSC pool with age, which would gradually lead to the senescence and impairment of the hfDSC pool (Figure 4K).

In order to gain insights into the mechanism by which β-catenin regulates HF senescence, we must first clarify the changes in HF mesenchyme which might underlie HF miniaturization. We then examined the expression of Cyr61, a marker of mesenchymal senescence (Jun and Lau, 2010; Abbasi et al., 2020), by using immunofluorescence staining. Significantly, in CD34:mTmG:β-cat^act^ mice, Cyr61 expression was found to be prominent in the DS and DP (Figure 4F). The corresponding elevated Cyr61 expression in HF mesenchyme induced by an activating mutation of β-catenin was similar to that seen in aged mice, indicating the existence of a semblable HF aging program. We also detected the expression of hfDSC-related markers. Remarkably, β-catenin-activated hfDSCs exhibited decreased expression of αSMA/ITGA5 as seen in the case of aged mice (Figures 4G,H), indicating the impairment of the hfDSC pool. These results collectively suggested that β-catenin activation in hfDSCs simulated the mesenchymal aging state and drove the HF aging process.

### 2.9 Forced Activation Enhancement of β-Catenin Signaling in hfDSCs Induces Senescent HFs Without DP Cells

CD34 is a marker of hair follicle stem cells and hfDSCs marker, we generated K15:mTmG:β-cat^act^ mice and found that β-catenin stabilization in K15^+^ hair follicle stem cells could not accelerate hair follicle senescence under the same experimental conditions. Similar to CD34^+^ cells, K15^+^ cells are situated in the bulge of telogen hair follicles. In some studies, researchers substitute K15CrePGR mice for CD34CrePGR mice. For example, Hsu et al. generated K15CrePGR:Rosa26-iDTR mice to solve scientific problems in CD34^+^ bulge stem cells (Hsu et al., 2011). So we also generated K15:mTmG:β-cat^act^ mice and found that senescence did not exist in these mice although they had destruction of hair follicle architecture (Supplementary Figures S8A–J). This suggested that β-catenin stabilization in hair follicle stem cells alone did not seem sufficient for the initiation of hair follicle senescence.

**FIGURE 5:**
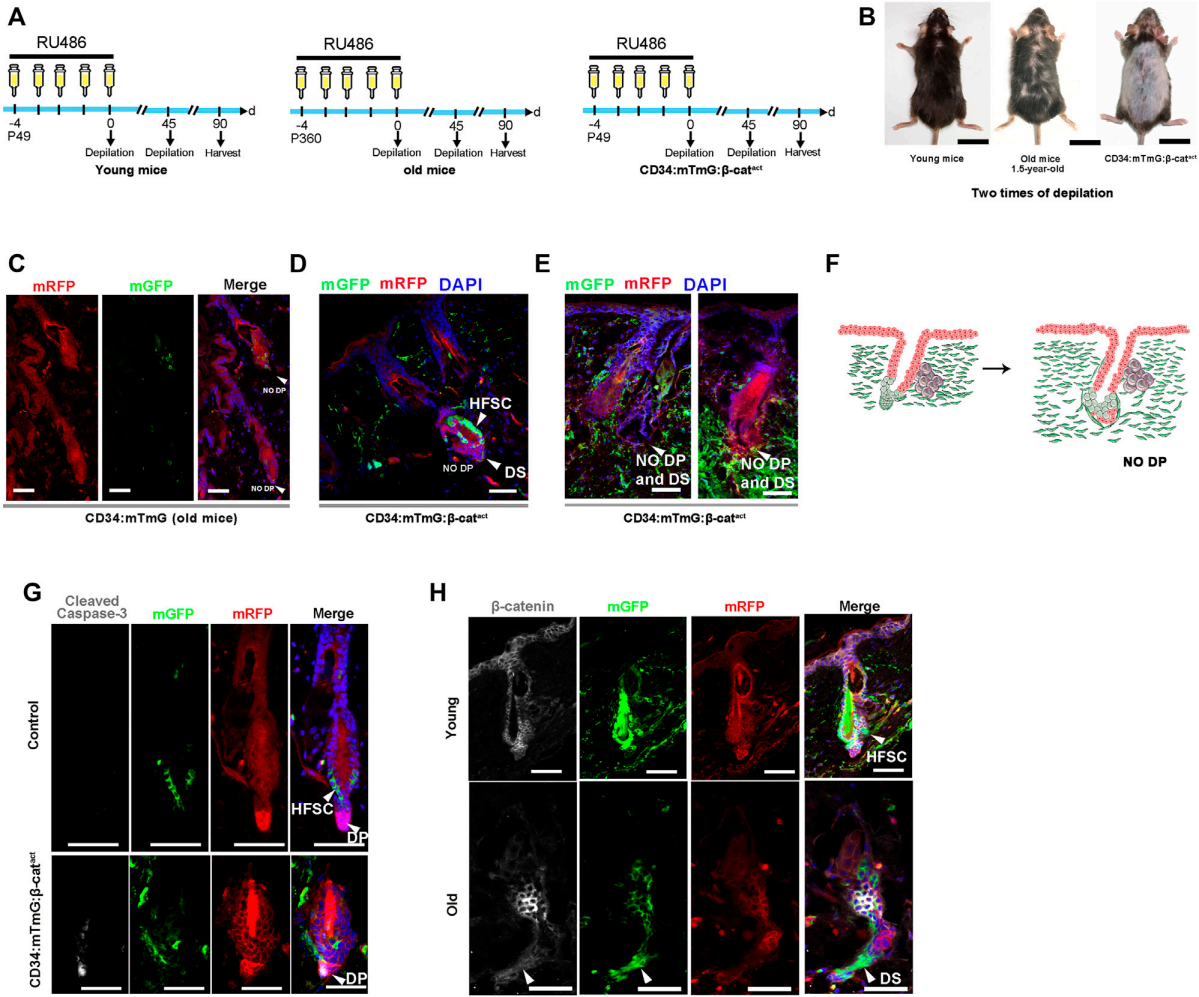
Ectopic activation of β-catenin leads to senescent HFs without DP cells. **(A)** Experimental timeline of RU486 injections and analysis in young CD34:mTmG, old CD34:mTmG, and CD34:mTmG:β-cat^act^ mice. **(B)** Young CD34:mTmG (left panel), old CD34:mTmG (middle panel), and CD34:mTmG:β-cat^act^ mice (right panel) that had undergone depilation-induced hair cycle twice. **(C)** Skin sections of 1.5-year-old CD34:mTmG mice. **(D,E)** CD34:mTmG:β-cat^act^ skin sections showing HFs without DP cells. Note that there was a ring of DS cells at the bottom of the HF, but the HFs did not contain DP. **(F)** Schematic illustration of the evolution of senescent HFs without DP cells. **(G)** CD34:mTmG:β-cat^act^ and CD34:mTmG skin immunostained for anti-cleaved caspase-3 antibody (white). Arrowhead highlights cleaved caspase-3 expression in DP cells. **(H)** CD34:mTmG:β-cat^act^ and CD34:mTmG skin were immunostained for β-catenin (white). Note elevated β-catenin expression in hfDSCs. Scale bars, 2 cm (B); 50 μm **(C–H)**.

To further investigate whether depletion of hfDSCs drives hair failure, we applied a repetitive depilation model to three groups of mice—young (7-week-old), aged (18-month-old), and β-catenin-activated (CD34:mTmG:β-cat^act^) mice. After three depilations, aged mice showed extensive hair thinning, whereas the hair density of young mice was not noticeably affected (Figure 5B). Surprisingly, upon the third depilation, hair loss was so conspicuous that there was almost no new hair growth in β-catenin-activated mice, which was much more severe than the phenotype observed in aged mice (Figure 5B).

Previously, it has been shown that the aging phenotype of hair is accompanied by a progressive decrease in the number of DP cells, and the HFs of aged mice do not even contain DP (Matsumura et al., 2016). We compared the skin slices of three groups of mice. Indeed, the incidence of DP was particularly low in the HFs of aged mice (Figure 5C). There was a ring of mGFP^+^ DS cells around the base of the HF, but no DP cells could be detected (Figure 5C; Supplementary Figure S5B). Astonishingly, HFs of β-catenin-activated mice also displayed a ring of mGFP^+^ DS cells surrounding the base of HFs, but almost no DP cells (Figures 5D,E). To determine whether HF mesenchyme in β-catenin-activated mice were undergoing apoptotic cell death, we stained CD34:mTmG:β-cat^act^ skin for cleaved caspase-3. Interestingly, prominent staining in DP cells was observed (Figure 5G), implying that DP cells were gradually apoptotic due to lack of hfDSCs, raising the specter that hfDSC exhaustion made it impossible for DP cells to be replenished and thus depleted, mimicking the aging phenotype of HFs.

Finally, we also verified the expression of β-catenin in aged hfDSCs. Compared with young mice, β-catenin expression was remarkably upregulated in hfDSCs of aged mice (Figure 5H), hence our data best fit the model in which β-catenin signaling might provokes HF senescence with the onset of advanced age to some extent.

## 3 Discussion

The ultimate goal of improving primary prevention and reducing the healthcare and socioeconomic impact of age-related diseases. With a continuous universal growing trend of a global aging society, the aging sequence and reverse-aging strategies that may mediate life-history trade-offs is undoubtedly become increasingly important for the medical and public health sector as the aging process is associated with progressive functional and structural deterioration, encompasses a main independent risk factor for vascular diseases, which is the leading cause of death worldwide, which associated with progressive functional and structural deterioration (Hamburg-Shields et al., 2015; Mastrogiannaki et al., 2016; Rognoni et al., 2016; Lim et al., 2018; Phan et al., 2020). HF senescence is the most noticeable common sign of the biological aging mechanism and demonstrative of human health, as it seems to prognosticate the systemic illness and prognosis which further increases the quality of life and to prevent age-related diseases.

Having a comprehensive understanding of HF senescence could yield important, novel insights into the molecular basis of skin aging and strategies for ameliorating aging-related skin diseases. Here, we show that Cre-mediated activation of β-catenin promotes the terminal differentiation of hfDSCs and HF aging. This excessive differentiation temporarily promotes hair growth, but ultimately led to a fairly significant aging phenotype, with hair appearing grayish and sparse. Our results indicate that proper β-catenin signaling is required for initiation and preservation of HF homeostasis, by tilting the molecular and cellular balance towards restoration of normal HF cycling.

Considerable heterogeneity was observed in dermal fibroblasts in response to Wnt/β-catenin signaling (Hamburg-Shields et al., 2015; Mastrogiannaki et al., 2016; Rognoni et al., 2016; Lim et al., 2018; Phan et al., 2020). Our study found that the homeostatic mechanism that governs hfDSCs is regulated by β-catenin signaling. In the wound experiment, somewhat unexpectedly, the wound environment elevated p-β-catenin expression in hfDSCs and induced emigration of CD34^+^ hfDSC progeny toward the DP. Anagen initiation is always accompanied by an increase in DP cell number, which is mainly achieved by the differentiation of hfDSCs into DP cells. This may partially explain why WIHA, a phenomenon concentrated near the wound edges. Previous studies presumed that this phenomenon was primarily due to the effect of wound macrophages on HF-SC activation (Wang et al., 2017; Rahmani et al., 2020). From the perspective of hfDSC differentiation, our studies provide an alternative explanation for WIHA which will be important toward understanding the extent of hfDSC contribution to DP homeostasis and function.

Based on the previous transcriptional analyses of hfDSCs carrying an activated allele of β-catenin (Tao et al., 2019), we postulated that β-catenin activation could induce acceleration of the hfDSC differentiation. To verify this, we performed a series of *in vivo* experiments. In addition to αSMACreERT2 transgenic mice that have been reported previously, in this particular study we also employed transgenic mice that express CD34CrePGR to target CD34^+^ hfDSCs. Remarkably, in the two transgenic mouse models expressing constitutive activated β-catenin, β-catenin signaling was found to promote hfDSC terminal differentiation. The withdrawal of hfDSC progeny from the DP at each degenerative stage to rebuild the hfDSC niche is essential to maintain a constant hfDSC population (Shin et al., 2020). In our study, β-catenin stabilization in CD34^+^ cells not only intensified depilation-induced anagen entry, but also triggers active spontaneous hair anagen to head telogen HFs. Significantly, hfDSC progeny lost their plasticity after terminal differentiation and therefore could not participate in recreating the SC pool, which led to the gradual depletion of hfDSCs and ultimately, an increase in DP cell death and aging-associated hair loss.

Recent work has highlighted that in canonical Wnt/β-catenin signaling, the secreted Wnt ligands bind with the Frizzled receptor, which further inactivates glycogen synthase kinase-3β (GSK-3β), which is an enzyme responsible for the ubiquitination-mediated degradation of β-catenin (Moon et al., 2004; Choi, 2020). Mechanistically, nuclear translocation of β-catenin is induced by the Wnt signal transduction pathway, stemmed in a concentration-dependent manner (Ridanpää et al., 2001). Currently, products with promising market prospects features that promotes HF regeneration as therapeutic approaches for alopecia, have also been identified as potential activators of Wnt/β-catenin signaling (Fakhraei Lahiji et al., 2018; Choi, 2020; Ji et al., 2021).

Hair loss postulated to be a process of HF atrophy, results from a progressive declination in the DP cell population (Elliott et al., 1999; Olsen et al., 2006; Matsumura et al., 2016). In the HFs, DP sends messages to its neighboring cell, keratinocytes, to instruct them to divide, and ultimately coordinate hair cycle so that new hair is continuously produced (Enshell-Seijffers et al., 2010). The lack of sufficient DP cells prevents the HFs from entering the next hair cycle, leading to irreversible hair loss (Elliott et al., 1999; Shin et al., 2020). Furthermore, studies have shown that the four microdomains within the DP regulates the activity of melanocytes via β-catenin signaling, to control hair color (Enshell-Seijffers et al., 2010). The close proximity of melanocyte progenitors to DP cells during all stages of hair cycle may also contribute to hfDSCs to act as cellular intermediate transmitting extrafollicular signals into the DP (Elliott et al., 1999; Shin et al., 2020). The maintenance of DP cells is highly dependent on hfDSCs, and the loss of hfDSCs prevents the production of new DP cells, so the growth of hair cannot be maintained (Shin et al., 2020).

In our study, we found that in DP cell apoptosis induced by hfDSC depletion eventually led to hair thinning, demonstrating that β-catenin stabilization in hfDSCs in young animals mimics the effect of aging in accelerating hair loss. Importantly, our study indicated that elevated Cyr61 expression in HF mesenchyme of β-catenin-activated mice was akin to the expression pattern of aged DP cells (Shin et al., 2020). Moreover, the depletion of the hfDSC pool led to the most severe result, which was that the DP cells gradually died out and resulted in DP-free HFs. This experimental model has thus provided a means to dissect the cellular and molecular basis for epidermal pathology. Further studies in humans are needed to establish the role of these pathways in clinical application.

Therapeutic interventions targeting hfDSCs has showed indication towards raising the intriguing possibility of treating skin aging which may also promote age-dependent changes in several organs, and their therapeutic properties may be an attractive approach to extend and influence healthy lifespan. Due to the limited availability of evidence-based hair restoration efficacy regimes that could induce and sustain disease remission, patients often turn to complementary and alternative medicine (CAM), whereby those modalities are often not regulated by the Food and Drug Administration (FDA) (Harada et al., 1999). Although there are existing FDA-approved medications, however it is still restricted due to adverse drug effects and poor prognosis towards this a psychologically debilitating disease (Hwang et al., 2021).

In conclusion, this study demonstrated the role of CD34^+^ hfDSCs in HF regeneration through *in vivo* lineage tracing, which further demonstrated that β-catenin signaling determines the differentiation of CD34^+^ hfDSCs into DP cells through β-catenin knockout and stabilization, and regulates the transformation of hair cycle and HF senescence. As heterogeneous epithelial SCs are compartmentalized not only for efficient homeostasis and regeneration, but also for defining patterned niches for specific epidermal-dermal interactions (Ito et al., 2007; Enshell-Seijffers et al., 2010; Tsai et al., 2014; Kaur et al., 2016; Tao et al., 2019). Therefore in our experiment, we demarcated the effects of CD34^+^ hfDSCs in HF senescence by accelerating their differentiation through β-catenin activation. Finally, our findings will be critical towards unraveling a better understanding towards the inductive process orchestrating the molecular mechanism of hfDSC differentiation.

Thus, we hope that our observations may pave the way to new avenues and broader insights into our etiology and pathogenesis of aging process beyond the HF, inspiring a driving development of targeted therapies with unprecedented clinical efficacy and future research to meet the unmet needs of aging biology.

## 4 Experimental Procedures

### 4.1 Mice

Rosa26-mTmG (stock no. 007576) strains were obtained from the Jackson Laboratory. αSMACreERT2 (Wendling et al., 2009) mice were a kind gift from Professor Pierre Chambon (IGBMC) and Dr. Gang Ma (Shanghai Jiao Tong University). Ctnnb1^tm1Mmt^ (Harada et al., 1999) mice were generous gifts from Dr. Liang Fang (Southern University of Science and Technology). We generate CD34-PGR promoter-driven CrePGR mouse model, refer to the study of Jiang et al. (2021). For additional experimental details please refer to the original article.

Mice of both genders were randomly selected to perform all lineage tracing experimentations with equal gender ratios. Group sizes of animal experiments included more than five animals in at least three independent experiments. The group size for animal studies was based on previous studies (Lee et al., 2008; Lee et al., 2012; Lee et al., 2017). All mice were bred and maintained in a specific pathogen-free (SPF) animal facility, with companion mice, and cages were placed under standard conditions (temperature 22 ± 2°C, humidity 55 ± 5%, 12:12 h light–dark cycle with lights on 6:00 a.m.), with free access to food and water. All of the animal procedures were performed with the approval of the Ethics Committee of Shenzhen Center for Disease and Prevention and Tsinghua Shenzhen International Graduate School. All of the animal experiments reported were in compliance with the ARRIVE guidelines (Percie du Sert et al., 2020) and were carried out in accordance with the relevant recommended guidelines and regulations made by the British Journal of Pharmacology (Lilley et al., 2020).

### 4.2 Genotyping of Mice

Mouse genomic DNA of mice was extracted from 2-mm mouse tails using a One Step Mouse Genotyping Kit (Vazyme Biotech Co., Ltd., Nanjin Jiangsu Province China). The chopped tails were added to mouse tissue lysis buffer (the ratio of lysis buffer to proteinase K was 50:1) and incubated for 30 min at 55°C in a water bath, followed by a 95°C water bath to inactivate protease K. The extracted samples were centrifuged at 12,000 rpm at 4°C for 5 min, and the supernatant was taken for PCR analysis. For detailed PCR conditions and primers, please refer to the Jackson Laboratory website (http://jaxmice.jax.org/strain/).

### 4.3 *In Vivo* Lineage Tracing

Intraperitoneal injection of 167 mg/kg tamoxifen (Sigma, 16.7 mg/ml in corn oil) was performed every 24 h to activate the CreER recombinase in αSMA:mTmG mice. For lineage tracing in CD34:mTmG mice, CD34 promoter-driven CrePGR recombinase was activated by intraperitoneal injection of 100 mg/kg RU486 (Sigma, 10 mg/ml in corn oil) every 24 h. Mice were treated with systemic intraperitoneal injections of RU486 for five consecutive days (refer to the schematic depiction for the specific time of injections administrated).

### 4.4 Cutaneous Wounding Experiments

Seven-week-old mice carrying a CD34CrePGR allele were treated with intraperitoneal inoculation of mifepristone (RU486) for five consecutive days. After administration, full-thickness wounds, 1.5 cm in diameter, were introduced into the dorsal skin at the appropriate time. Different experiments were conducted at different time points to create wounds, as shown in the schematic diagram of each experiment. Twenty days post wounding, skin samples containing the central wound bed and peripheral HFs were harvested for observation. In the case of the linear incision, the wound was divided into three equal width longitudinal strips perpendicular to the direction of the incision.

### 4.5 Tissue Preparation and Immunofluorescence Staining

Routine staining in tissue processing, also known as hematoxylin and eosin stain (H&E) and immunostaining were employed according to published protocols. Skin samples were fixed in 4% PFA for 8 h at room temperature (RT). Subsequently, skin tissues were soaked in PBS for 8 h and then dehydrated with PBS solution consisting of 30% sucrose for 8 h. The skin tissues were then embedded in optimal cutting temperature (OCT) compound. Sections were then subsequently snap frozen and sectioned (10-μm-thick). Sections were prepared with a cryostat (Leica, Germany) and samples stored at −80°C until analyzed.

The frozen skin samples were then placed at RT for 10 min and then soaked in PBS for 10 min. After permeabilizing and blocking in 10% goat serum (solution containing 3% BSA and 0.5% Triton X-100) at 37°C for 60 min, sections were incubated overnight with a primary antibody at 4°C (dissolved in PBS solution containing 1% BSA). The primary antibodies used were CD34 (Invitrogen, MA5-32059, 1:50), ITGA5 (Abcam, ab150361, 1:50), αSMA (Abcam, ab32575, 1:500), phospho-β-catenin (Cell Signaling, 5651, 1:100), β-catenin (Sigma, C2206, 1:150) and cleaved caspase-3 (Beyotime, AC033, 1:400). The sections were washed with PBS three times, followed by incubation with secondary antibody (Jackson ImmunoResearch, 111-605-003, 1:200) at 37°C for 60 min and washing with PBS three times. Nuclei were stained with DAPI (Beijing Solarbio Science & Technology Co., Ltd., Shenzhen Guangzhou Province China). All images were captured using a Zeiss confocal microscope.

### 4.6 Quantitative and Statistical Analysis

In this study, all experiments had roughly equal numbers of male and female mice, with no significant difference between the sexes. The number of mice in all independent experiments was more than three, Results displayed in figures were representative patterns from at least three animals treated independently. Differences between groups were determined by unpaired Student’s t-test, and genotypes were identified prior to the experiment. Data are presented as mean ± standard mean of error (mean ± SEM). *p* < 0.05 was considered statistically significant (^∗^ *p* < 0.05, ^∗∗^ *p* < 0.01, ^∗∗∗^ *p* < 0.001). Experiment results were analyzed using GraphPad Prism 8.0.2. Figures were prepared using Adobe Photoshop 2021 and Adobe Illustrator 2021.

### 4.7 HE Staining

The HE staining procedure followed the BJP guidelines (Alexander et al., 2018) and previous study (Chen et al., 2019). Fresh frozen sections were thawed at RT and washed with PBS for 10 min. Sections were stained with hematoxylin for 5 min, and rinsed in tap water. Sections were rinsed in 1% hydrochloric acid in alcohol for 30 s, and in tap water for 15 min. Sections were counterstained with eosin for 2 min and washed with tap water. Sections were dehydrated through an alcohol gradient series and soaked in xylene twice. Slides were sealed with neutral gum and stored at RT.

### 4.8 CD34-CrePR1-P2A-tdTomato Mouse Generation

To further assess the characteristics of CD34^+^ mesenchymal cells, we attempted to create a CD34-CrePGR mouse line that expresses CrePGR under the control of the CD34 promoter. Fortunately, we successfully generated a CD34-CrePR1-P2A-tdTomato mouse model in which tdTomato fluorescent protein is selectively expressed in CD34-expressing cells. CD34-CrePR1-P2A-tdTomato mouse is self-designed and designed by biocyto Company Limited.

CD34-CrePR1-P2A-tdTomato mice was RU486-inducible Cre recombinase and generated by conventional embryonic stem cell gene targeting methods. Targeting vector was designed to insert a cDNA encoding a Cre recombinase-progesterone receptor 1 (CrePR1) fusion protein into ATG of gene CD34 (Plus strand on chromosome 1) The plasmid structure contains 5′ homologous arm (∼2 kb), Knock-In cassette (∼2 kb), 3′ homologous arm (∼2 kb). T7 promoter plasmid vector (ATG-CrePR1-P2A-tdtomato-WPRE-bGH polyA)and *in vitro* transcription was then linearized and electroporated into mouse embryonic stem cells. The selected embryonic stem cells were injected into the blastocyst to obtain the selected mice. The F0 mice were chimeras. Therefore, the F0 genotype was obtained by genotyping F0 mouse tail. Allelic mice were bred under the background of C57. Finally, F1 generation positive mice were generated and Southern blot analysis.

## Data Availability

The original contributions presented in the study are included in the article/Supplementary Material, further inquiries can be directed to the corresponding authors.

## Supplementary Material

Supplementary Figure 1 HFs from CD34:mTmG mice in the first and second depilation-induced hair cycle. **(A–C)** Telogen **(A)**, anagen **(B)** and catagen **(C)** HFs during the first depilation-induced hair cycle immunostained with ITGA5. **(D,E)** Immunostaining of telogen **(D)** and anagen **(E)** HFs for ITGA5. Scale bars, 50 μm.

Supplementary Figure 2 Fate of CD34^+^ adult mesenchymal cells in wound proximity. **(A)** Experimental timeline of the lineage tracing strategy and wounding experiments in adult CD34:mTmG mice. **(B)** Telogen HFs at the wound edge (left panel). Arrowhead highlights the expansion of hfDSCs. Sections of HFs close to a wound (right panel). Note DS cells with a very high labeling efficiency. **(C)** HFs close to a wound showing mGFP-labeled cells within DP above Auber’s line. Arrowhead indicates the expression of ITGA5 within DP. mRFP, membrane red fluorescent protein. **(D)** Proposed model showing CD34-lineage mesenchymal cells (green) within *de novo* HFs and peripheral HFs. PF, peripheral follicles. **(E)** Immunostaining for p-β-catenin in hair follicles adjacent to wounds. Note DC cells with elevated p-β-catenin expression. **(F)** Skin sections from CD34:mTmG mice immunostained with p-β-catenin. Scale bars, 50 μm.

Supplementary Figure 3 Representative HE images. **(A,B)** Skin sections of CD34:mTmG:β-cat^act^ and mTmG:β-cat^act^ mice at 0 day. **(C,D)** Skin sections of CD34:mTmG:β-cat^act^ and mTmG:β-cat^act^ mice at 10 days post depilation. **(E,F)** Skin sections of CD34:mTmG:β-cat^act^ and mTmG:β-cat^act^ mice at 25 days post depilation. **(G,H)** Skin sections of αSMA:mTmG:β-cat^act^ and mTmG:β-cat^act^ mice at 10 days post depilation. **(I,J)** Skin sections of αSMA:mTmG:β-cat^act^ and mTmG:β-cat^act^ mice at 25 days post depilation. Scale Bar: 250 μm.

Supplementary Figure 4 Skin sections of CD34:mTmG:β-cat^act^ mice immunostained with β-catenin. **(A,B)** Expression of β-catenin (white) was observed by immunofluorescence in the HFs of CD34:mTmG:β-catact (upper panel) mice. Note Arrowheads indicate nuclear β-catenin expression.

Supplementary Figure 5 CD34:mTmG:β-cat^act^ and αSMA:mTmG: β-cat^act^ mice image analyses data quantified. **(A)** Quantitation of mGFP + cells within DP ratio in CD34:mTmG: β-cat^act^ and CD34:mTmG during the 2nd hair follicle cycle after depliation. **(B)** Quantitation of no DP ratio in CD34:mTmG: β-cat^act^ and CD34:mTmG during the 3rd hair follicle cycle after depliation. **(C)** Representative images showing hair follicles in αSMA:mTmG: β-cat^act^ and αSMA:mTmG mice at different stages of the second depilation-induced hair cycle. **(D)** Quantitation of mGFP + cells within DP ratio in CD34:mTmG: β-cat^act^ and CD34:mTmG during the 2nd hair follicle cycle after depliation. Scale Bar, 50 μm. *n* > 3 mice. ^∗∗∗^ *p* < 0.005.

Supplementary Figure 6 β-catenin stabilization results in accelerated differentiation of αSMA^+^ hfDSCs into DP cells. **(A–C)** Representative images showing HFs in αSMA:mTmG:β-cat^act^ mice at different stages of the first depilation-induced hair cycle. **(D)** Schematic summary of the fate of αSMA-lineage cells (green) during the first depilation-induced hair cycle. **(E,F)** Representative images showing HFs in αSMA:mTmG:β-cat^act^ mice at different stages of the second depilation-induced hair cycle. **(G)** Schematic summary of the fate of αSMA-lineage cells (green) during the second depilation-induced hair cycle. Scale bars, 50 μm.

Supplementary Figure 7 CD34^+^ mesenchymal cells give rise to DP and DS cells upon transplantation. **(A,B)** Skin tissues from CD34:mTmG **(A)** and CD34:mTmG:β-cat^act^ **(B)** mice 1 day postinjection. Dotted lines represent adipose layer. **(C,D)** Skin slices from CD34:mTmG **(C)** and CD34:mTmG:β-cat^act^ **(D)** mice 7 days postinjection. **(E,F)** Dorsal skin sections of CD34:mTmG **(E)** and CD34:mTmG:β-cat^act^ **(F)** mice 20 days postinjection. Arrowhead indicates mGFP-labeled DP. Scale bars, 50 μm.

Supplementary Figure 8 K15:β-cat^act^ mice did not show aging phenotype. **(A)** Representative pictures of K15:β-cat^act^ mice and control β-cat^act^ littermates at 25 days post depilation. There was no noticeable thinning of hair in K15:β-cat^act^ mice. **(B,C)** Representative HE images of skin sections from K15:β-cat^act^ and β-cat^act^ mice at 10 days post depilation. **(D,E)** Representative HE images of skin sections from K15:β-cat^act^ and β-cat^act^ mice at 25 days post depilation. **(F,G)** Representative HE images of skin sections from K15:β-cat^act^ and β-cat^act^ mice at 25 days post depilation. **(H)** Representative pictures of K15:β-cat^act^ mice and control β-cat^act^ littermates at 60 days post second depilation. **(I,J)** Representative HE images of skin sections from K15:β-cat^act^ and β-cat^act^ mice at 60 days post second depilation. There was no noticeable thinning of hair in K15:β-cat^act^ mice. Scale bar, 250 μm.

Supplementary Figure 9 Representative pictures of after several depilation in old mice (Left), Left. Old mice showed white hair aging phenotype after several depilation. Right. Old mice showed hair less aging phenotype after several depilation.

## References

[B1] Aamar E. Avigad Laron E. Asaad W. Harshuk-Shabso S. Enshell-Seijffers D. (2021). Hair-Follicle Mesenchymal Stem Cell Activity during Homeostasis and Wound Healing. J. Invest. Dermatol. 141, 2797–2807. 10.1016/j.jid.2021.05.023 34166673

[B2] Abbasi S. Sinha S. Labit E. Rosin N. L. Yoon G. Rahmani W. (2020). Distinct Regulatory Programs Control the Latent Regenerative Potential of Dermal Fibroblasts during Wound Healing. Cell Stem Cell 27, 396–412. 10.1016/j.stem.2020.07.008 32755548

[B3] Alexander S. P. H. Roberts R. E. Broughton B. R. S. Sobey C. G. George C. H. Stanford S. C. (2018). Goals and Practicalities of Immunoblotting and Immunohistochemistry: A Guide for Submission to the British Journal of Pharmacology. Br. J. Pharmacol. 175, 407–411. 10.1111/bph.14112 29350411 PMC5773976

[B4] Argyris T. S. (1956). The Effect of Wounds on Adjacent Growing or Resting Hair Follicles in Mice. AMA Arch. Pathol. 61, 31–36. 13275201

[B5] Biernaskie J. Paris M. Morozova O. Fagan B. M. Marra M. Pevny L. (2009). SKPs Derive from Hair Follicle Precursors and Exhibit Properties of Adult Dermal Stem Cells. Cell Stem Cell 5, 610–623. 10.1016/j.stem.2009.10.019 19951689 PMC2828150

[B6] Blanpain C. Lowry W. E. Geoghegan A. Polak L. Fuchs E. (2004). Self-renewal, Multipotency, and the Existence of Two Cell Populations within an Epithelial Stem Cell Niche. Cell 118, 635–648. 10.1016/j.cell.2004.08.012 15339667

[B7] Chen H. Wang X. Chen Y. Han J. Kong D. Zhu M. (2019). Pten Loss in Lgr5+ Hair Follicle Stem Cells Promotes SCC Development. Theranostics 9, 8321–8331. 10.7150/thno.35467 31754399 PMC6857063

[B8] Chi W. Wu E. Morgan B. A. (2013). Dermal Papilla Cell Number Specifies Hair Size, Shape and Cycling and its Reduction Causes Follicular Decline. Development 140, 1676–1683. 10.1242/dev.090662 23487317 PMC3621486

[B9] Choi B. Y. (2020). Targeting Wnt/β-Catenin Pathway for Developing Therapies for Hair Loss. Int. J. Mol. Sci. 21, 4915. 10.3390/ijms21144915 PMC7404278 32664659

[B10] Courtois M. Loussouarn G. Hourseau C. Grollier J. F. (1995). Ageing and Hair Cycles. Br. J. Dermatol. 132, 86–93. 10.1111/j.1365-2133.1995.tb08630.x 7756156

[B11] Elliott K. Messenger A. G. Stephenson T. J. (1999). Differences in Hair Follicle Dermal Papilla Volume Are Due to Extracellular Matrix Volume and Cell Number: Implications for the Control of Hair Follicle Size and Androgen Responses. J. Invest. Dermatol. 113, 873–877. 10.1046/j.1523-1747.1999.00797.x 10594724

[B12] Enshell-Seijffers D. Lindon C. Kashiwagi M. Morgan B. A. (2010). β-Catenin Activity in the Dermal Papilla Regulates Morphogenesis and Regeneration of Hair. Develop. Cel 18, 633–642. 10.1016/j.devcel.2010.01.016 PMC2893731 20412777

[B13] Fakhraei Lahiji S. Seo S. H. Kim S. Dangol M. Shim J. Li C. G. (2018). Transcutaneous Implantation of Valproic Acid-Encapsulated Dissolving Microneedles Induces Hair Regrowth. Biomaterials 167, 69–79. 10.1016/j.biomaterials.2018.03.019 29554482

[B14] Geyfman M. Andersen B. (2010). Clock Genes, Hair Growth and Aging. Aging 2, 122–128. 10.18632/aging.100130 20375466 PMC2871241

[B15] Hagner A. Shin W. Sinha S. Alpaugh W. Workentine M. Abbasi S. (2020). Transcriptional Profiling of the Adult Hair Follicle Mesenchyme Reveals R-Spondin as a Novel Regulator of Dermal Progenitor Function. Iscience 23, 101019. 10.1016/j.isci.2020.101019 32289736 PMC7155209

[B16] Hamburg-Shields E. Dinuoscio G. J. Mullin N. K. Lafyatis R. Atit R. P. (2015). Sustained β-catenin Activity in Dermal Fibroblasts Promotes Fibrosis by Up-Regulating Expression of Extracellular Matrix Protein-Coding Genes. J. Pathol. 235, 686–697. 10.1002/path.4481 25385294 PMC4357547

[B17] Harada N. Tamai Y. Ishikawa T. Sauer B. Takaku K. Oshima M. (1999). Intestinal Polyposis in Mice with a Dominant Stable Mutation of the Beta -catenin Gene. EMBO J. 18, 5931–5942. 10.1093/emboj/18.21.5931 10545105 PMC1171659

[B18] Harrison D. E. Archer J. R. (1988). Biomarkers of Aging: Tissue Markers. Future Research Needs, Strategies, Directions and Priorities. Exp. Gerontol. 23, 309–321. 10.1016/0531-5565(88)90034-4 3197782

[B19] He J. Duan H. Xiong Y. Zhang W. Zhou G. Cao Y. (2013). Participation of CD34-Enriched Mouse Adipose Cells in Hair Morphogenesis. Mol. Med. Rep. 7, 1111–1116. 10.3892/mmr.2013.1307 23404453

[B20] Hoogduijn M. J. Gorjup E. Genever P. G. (2006). Comparative Characterization of Hair Follicle Dermal Stem Cells and Bone Marrow Mesenchymal Stem Cells. Stem Cell Develop. 15, 49–60. 10.1089/scd.2006.15.49 16522162

[B21] Hsu Y.-C. Pasolli H. A. Fuchs E. (2011). Dynamics between Stem Cells, Niche, and Progeny in the Hair Follicle. Cell 144, 92–105. 10.1016/j.cell.2010.11.049 21215372 PMC3050564

[B60] Hwang J-H Lee H-Y Chung K. B. Lee H. J. Kim J. Song K. (2021). Non-Thermal Atmospheric Pressure Plasma Activates Wnt/β-Catenin Signaling in Dermal Papilla Cells. Sci. Rep. 11, 16125. 10.1038/s41598-021-95650-y PMC8352944 34373562

[B22] Ito M. Yang Z. Andl T. Cui C. Kim N. Millar S. E. (2007). Wnt-dependent De Novo Hair Follicle Regeneration in Adult Mouse Skin after Wounding. Nature 447, 316–320. 10.1038/nature05766 17507982

[B23] Ji S. Zhu Z. Sun X. Fu X. (2021). Functional Hair Follicle Regeneration: an Updated Review. Sig. Transduct. Target. Ther. 6, 66. 10.1038/s41392-020-00441-y PMC7886855 33594043

[B24] Jiang L. Chen T. Sun S. Wang R. Deng J. Lyu L. (2021). Nonbone Marrow CD34+ Cells Are Crucial for Endothelial Repair of Injured Artery. Circ. Res. 129, e146–e165. 10.1161/CIRCRESAHA.121.319494 34474592

[B25] Joost S. Annusver K. Jacob T. Sun X. Dalessandri T. Sivan U. (2020). The Molecular Anatomy of Mouse Skin during Hair Growth and Rest. Cell Stem Cell 26, 441–457. 10.1016/j.stem.2020.01.012 32109378

[B26] Joshi S. S. Tandukar B. Pan L. Huang J. M. Livak F. Smith B. J. (2019). CD34 Defines Melanocyte Stem Cell Subpopulations with Distinct Regenerative Properties. Plos Genet. 15, e1008034. 10.1371/journal.pgen.1008034 31017901 PMC6481766

[B27] Jun J.-I. Lau L. F. (2010). The Matricellular Protein CCN1 Induces Fibroblast Senescence and Restricts Fibrosis in Cutaneous Wound Healing. Nat. Cel Biol. 12, 676–685. 10.1038/ncb2070 PMC2919364 20526329

[B28] Kaur A. Webster M. R. Weeraratna A. T. (2016). In the Wnt-Er of Life: Wnt Signalling in Melanoma and Ageing. Br. J. Cancer 115, 1273–1279. 10.1038/bjc.2016.332 27764844 PMC5129830

[B29] Lee S.-H. Chung M.-K. Sohn Y.-J. Lee Y.-S. Kang K.-S. (2008). Human Hair Follicle Cells with the Cell Surface Marker CD34 Can Regenerate New Mouse Hair Follicles and Located in the Outer Root Sheath of Immunodeficient Nude Mice. Int. J. Stem Cell 1, 70–81. 10.15283/ijsc.2008.1.1.70 PMC4021778 24855511

[B30] Lee S.-H. Yoon J. Shin S. H. Zahoor M. Kim H. J. Park P. J. (2012). Valproic Acid Induces Hair Regeneration in Murine Model and Activates Alkaline Phosphatase Activity in Human Dermal Papilla Cells. PLoS One 7, e34152. 10.1371/journal.pone.0034152 22506014 PMC3323655

[B31] Lee S.-H. Seo S. H. Lee D.-H. Pi L.-Q. Lee W.-S. Choi K.-Y. (2017). Targeting of CXXC5 by a Competing Peptide Stimulates Hair Regrowth and Wound-Induced Hair Neogenesis. J. Invest. Dermatol. 137, 2260–2269. 10.1016/j.jid.2017.04.038 28595998

[B32] Lilley E. Stanford S. C. Kendall D. E. Alexander S. P. H. Cirino G. Docherty J. R. (2020). ARRIVE 2.0 and the British Journal of Pharmacology: Updated Guidance for 2020. Br. J. Pharmacol. 177, 3611–3616. 10.1111/bph.15178 32662875 PMC7393193

[B33] Lim C. H. Sun Q. Ratti K. Lee S.-H. Zheng Y. Takeo M. (2018). Hedgehog Stimulates Hair Follicle Neogenesis by Creating Inductive Dermis during Murine Skin Wound Healing. Nat. Commun. 9, 4903. 10.1038/s41467-018-07142-9 30464171 PMC6249328

[B34] Lin W. H. Xiang L. J. Shi H. X. Zhang J. Jiang L. P. Cai P. T. (2015). Fibroblast Growth Factors Stimulate Hair Growth through β-catenin and Shh Expression in C57BL/6 Mice. Biomed. Res. Int. 2015, 730139. 10.1155/2015/730139 25685806 PMC4313060

[B35] Liu L. Cheung T. H. Charville G. W. Hurgo B. M. C. Leavitt T. Shih J. (2013). Chromatin Modifications as Determinants of Muscle Stem Cell Quiescence and Chronological Aging. Cel Rep. 4, 189–204. 10.1016/j.celrep.2013.05.043 PMC4103025 23810552

[B36] López-Otín C. Blasco M. A. Partridge L. Serrano M. Kroemer G. (2013). The Hallmarks of Aging. Cell 153, 1194–1217. 10.1016/j.cell.2013.05.039 23746838 PMC3836174

[B37] Mastrogiannaki M. Lichtenberger B. M. Reimer A. Collins C. A. Driskell R. R. Watt F. M. (2016). β-Catenin Stabilization in Skin Fibroblasts Causes Fibrotic Lesions by Preventing Adipocyte Differentiation of the Reticular Dermis. J. Invest. Dermatol. 136, 1130–1142. 10.1016/j.jid.2016.01.036 26902921 PMC4874948

[B38] Matsumura H. Mohri Y. Binh N. T. Morinaga H. Fukuda M. Ito M. (2016). Hair Follicle Aging Is Driven by Transepidermal Elimination of Stem Cells via COL17A1 Proteolysis. Science 351, aad4395. 10.1126/science.aad4395 26912707

[B39] McElwee K. J. Kissling S. Wenzel E. Huth A. Hoffmann R. (2003). Cultured Peribulbar Dermal Sheath Cells Can Induce Hair Follicle Development and Contribute to the Dermal Sheath and Dermal Papilla. J. Invest. Dermatol. 121, 1267–1275. 10.1111/j.1523-1747.2003.12568.x 14675169

[B40] Mok K.-W. Saxena N. Heitman N. Grisanti L. Srivastava D. Muraro M. J. (2019). Dermal Condensate Niche Fate Specification Occurs Prior to Formation and Is Placode Progenitor Dependent. Develop. Cel 48, 32–48. 10.1016/j.devcel.2018.11.034 PMC6370312 30595537

[B41] Moon R. T. Kohn A. D. Ferrari G. V. D. Kaykas A. (2004). WNT and β-catenin Signalling: Diseases and Therapies. Nat. Rev. Genet. 5, 691–701. 10.1038/nrg1427 15372092

[B42] Olsen E. A. Hordinsky M. Whiting D. Stough D. Hobbs S. Ellis M. L. (2006). The Importance of Dual 5α-Reductase Inhibition in the Treatment of Male Pattern Hair Loss: Results of a Randomized Placebo-Controlled Study of Dutasteride versus Finasteride. J. Am. Acad. Dermatol. 55, 1014–1023. 10.1016/j.jaad.2006.05.007 17110217

[B43] Percie du Sert N. Hurst V. Ahluwalia A. Alam S. Avey M. T. Baker M. (2020). The ARRIVE Guidelines 2.0: Updated Guidelines for Reporting Animal Research. Plos Biol. 18, e3000410. 10.1371/journal.pbio.3000410 32663219 PMC7360023

[B44] Phan Q. M. Fine G. M. Salz L. Herrera G. G. Wildman B. Driskell I. M. (2020). Lef1 Expression in Fibroblasts Maintains Developmental Potential in Adult Skin to Regenerate Wounds. Elife 9, e60066. 10.7554/eLife.60066 32990218 PMC7524549

[B45] Rabbani P. Takeo M. Chou W. Myung P. Bosenberg M. Chin L. (2011). Coordinated Activation of Wnt in Epithelial and Melanocyte Stem Cells Initiates Pigmented Hair Regeneration. Cell 145, 941–955. 10.1016/j.cell.2011.05.004 21663796 PMC3962257

[B46] Rahmani W. Abbasi S. Hagner A. Raharjo E. Kumar R. Hotta A. (2014). Hair Follicle Dermal Stem Cells Regenerate the Dermal Sheath, Repopulate the Dermal Papilla, and Modulate Hair Type. Develop. Cel 31, 543–558. 10.1016/j.devcel.2014.10.022 25465495

[B47] Rahmani W. Sinha S. Biernaskie J. (2020). Immune Modulation of Hair Follicle Regeneration. NPJ Regen. Med. 5, 9. 10.1038/s41536-020-0095-2 32411394 PMC7214459

[B48] Ridanpää M. Van Eenennaam H. Pelin K. Chadwick R. Johnson C. Yuan B. (2001). Mutations in the RNA Component of RNase MRP Cause a Pleiotropic Human Disease, Cartilage-Hair Hypoplasia. Cell 104, 195–203. 10.1016/s0092-8674(01)00205-7 11207361

[B49] Rinkevich Y. Walmsley G. G. Hu M. S. Maan Z. N. Newman A. M. Drukker M. (2015). Skin Fibrosis. Identification and Isolation of a Dermal Lineage with Intrinsic Fibrogenic Potential. Science 348, aaa2151. 10.1126/science.aaa2151 25883361 PMC5088503

[B50] Rognoni E. Gomez C. Pisco A. O. Rawlins E. L. Simons B. D. Watt F. M. (2016). Inhibition of β-catenin Signalling in Dermal Fibroblasts Enhances Hair Follicle Regeneration during Wound Healing. Development 143, 2522–2535. 10.1242/dev.131797 27287810 PMC4958333

[B51] Salzer M. C. Lafzi A. Berenguer-Llergo A. Youssif C. Castellanos A. Solanas G. (2018). Identity Noise and Adipogenic Traits Characterize Dermal Fibroblast Aging. Cell 175, 1575–1590. 10.1016/j.cell.2018.10.012 30415840

[B52] Shin W. Rosin N. L. Sparks H. Sinha S. Rahmani W. Sharma N. (2020). Dysfunction of Hair Follicle Mesenchymal Progenitors Contributes to Age-Associated Hair Loss. Develop. Cel 53, 185–198. 10.1016/j.devcel.2020.03.019 32315612

[B53] Shook B. A. Wasko R. R. Rivera-Gonzalez G. C. Salazar-Gatzimas E. López-Giráldez F. Dash B. C. (2018). Myofibroblast Proliferation and Heterogeneity Are Supported by Macrophages during Skin Repair. Science 362, eaar2971. 10.1126/science.aar2971 30467144 PMC6684198

[B54] Tao Y. Yang Q. Wang L. Zhang J. Zhu X. Sun Q. (2019). β-Catenin Activation in Hair Follicle Dermal Stem Cells Induces Ectopic Hair Outgrowth and Skin Fibrosis. J. Mol. Cel Biol. 11, 26–38. 10.1093/jmcb/mjy032 29771334

[B55] Trempus C. S. Morris R. J. Bortner C. D. Cotsarelis G. Faircloth R. S. Reece J. M. (2003). Enrichment for Living Murine Keratinocytes from the Hair Follicle Bulge with the Cell Surface Marker CD34. J. Invest. Dermatol. 120, 501–511. 10.1046/j.1523-1747.2003.12088.x 12648211

[B56] Tsai S.-Y. Sennett R. Rezza A. Clavel C. Grisanti L. Zemla R. (2014). Wnt/β-catenin Signaling in Dermal Condensates Is Required for Hair Follicle Formation. Develop. Biol. 385, 179–188. 10.1016/j.ydbio.2013.11.023 24309208 PMC3933391

[B57] Wang X. Chen H. Tian R. Zhang Y. Drutskaya M. S. Wang C. (2017). Macrophages Induce AKT/β-catenin-dependent Lgr5+ Stem Cell Activation and Hair Follicle Regeneration through TNF. Nat. Commun. 8, 14091. 10.1038/ncomms14091 28345588 PMC5378973

[B58] Wendling O. Bornert J.-M. Chambon P. Metzger D. (2009). Efficient Temporally-Controlled Targeted Mutagenesis in Smooth Muscle Cells of the Adult Mouse. Genesis 47, 14–18. 10.1002/dvg.20448 18942088

[B59] Wu Z. Yang L. Cai L. Zhang M. Cheng X. Yang X. (2007). Detection of Epithelial to Mesenchymal Transition in Airways of a Bleomycin Induced Pulmonary Fibrosis Model Derived from an α-smooth Muscle Actin-Cre Transgenic Mouse. Respir. Res. 8, 1. 10.1186/1465-9921-8-1 17207287 PMC1781437

