# Supplementary figures and images for: β-Catenin Signaling Evokes Hair Follicle Senescence by Accelerating the Differentiation of Hair Follicle Mesenchymal Progenitors
Source: Front Cell Dev Biol. 2022 Apr 4;10:839519. doi: 10.3389/fcell.2022.839519 (PMC9037041; doi:10.3389/fcell.2022.839519)

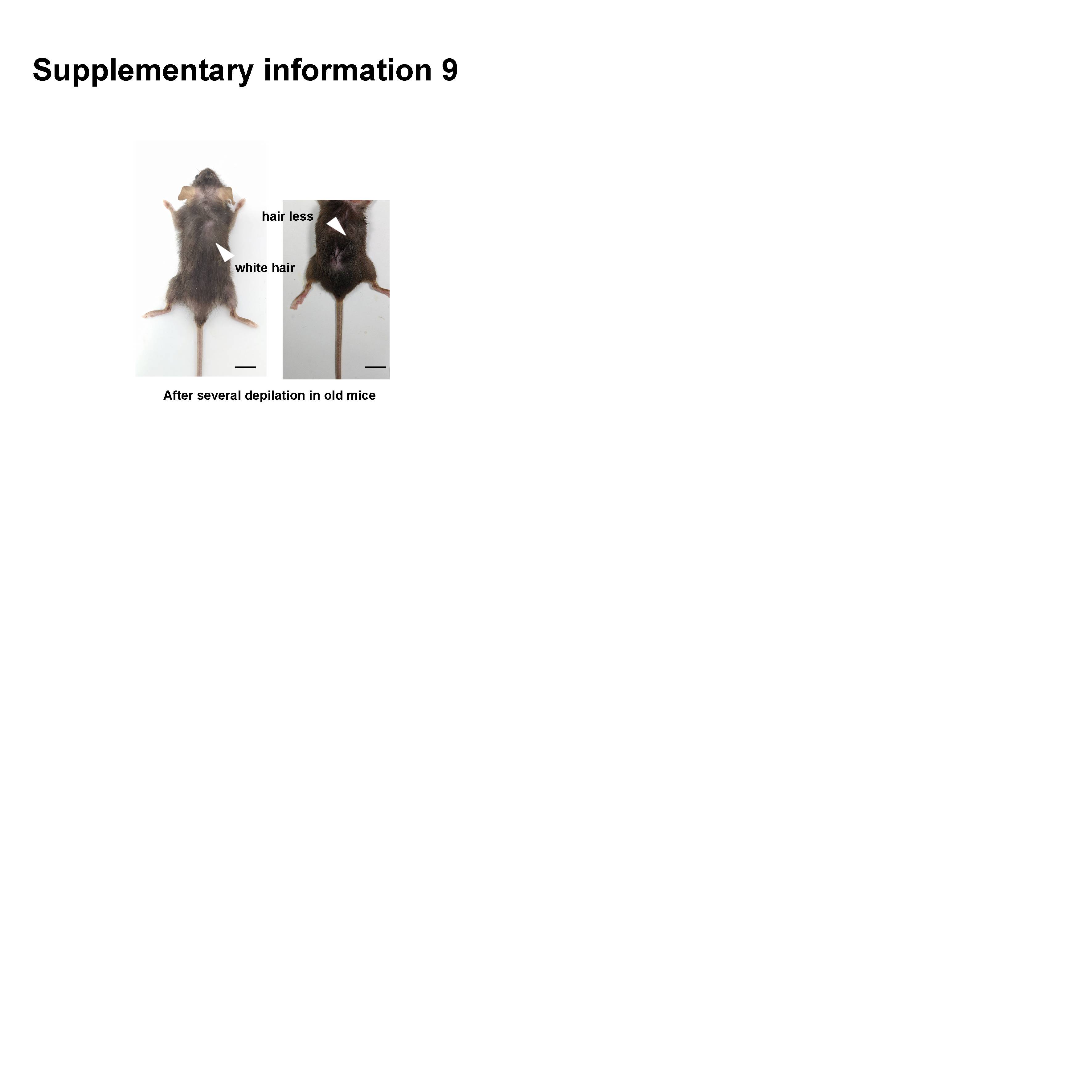

Supplement: Supplementary file 1 [file Image9.JPEG]

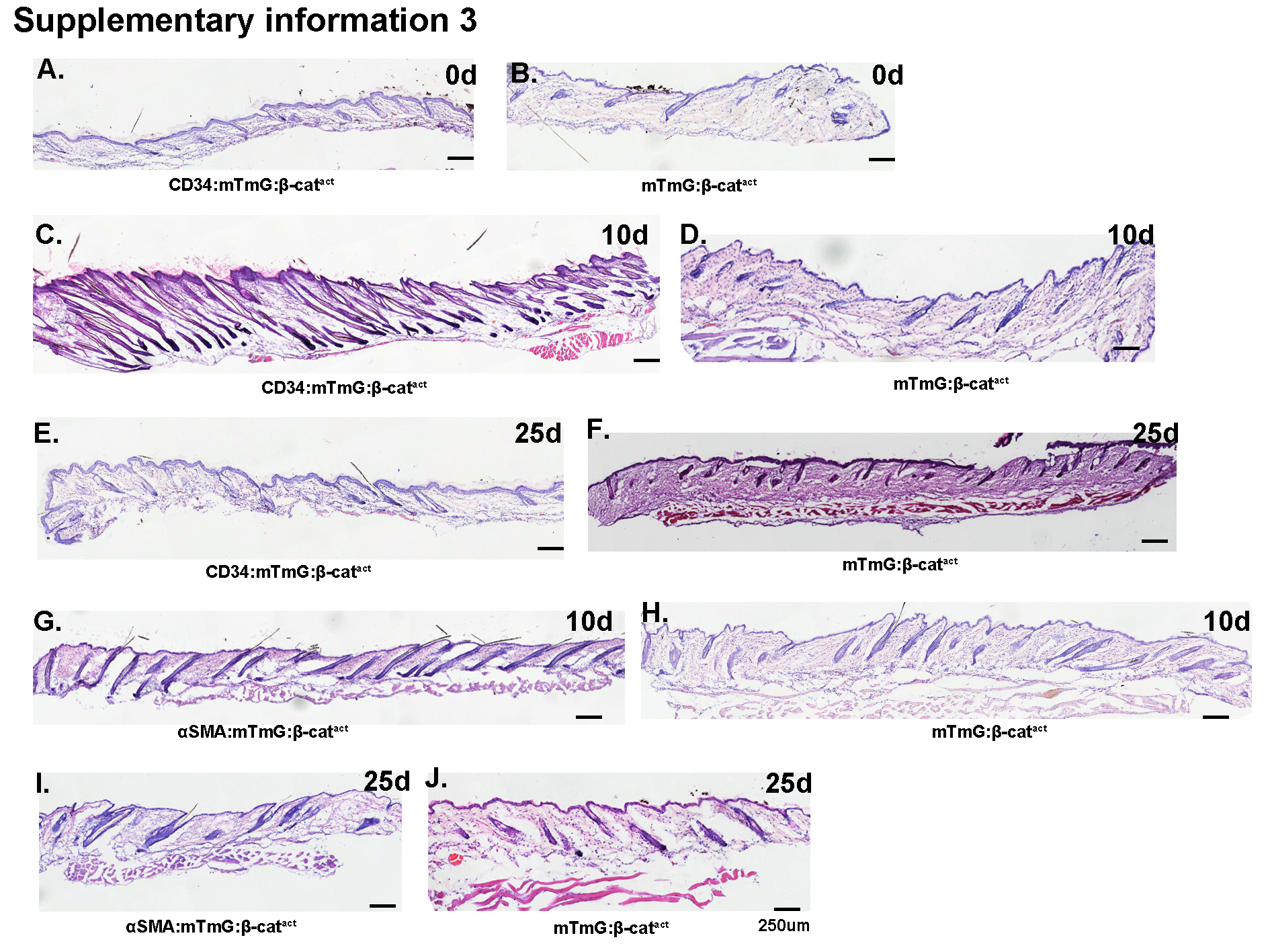

Supplement: Supplementary file 2 [file Image3.TIF]

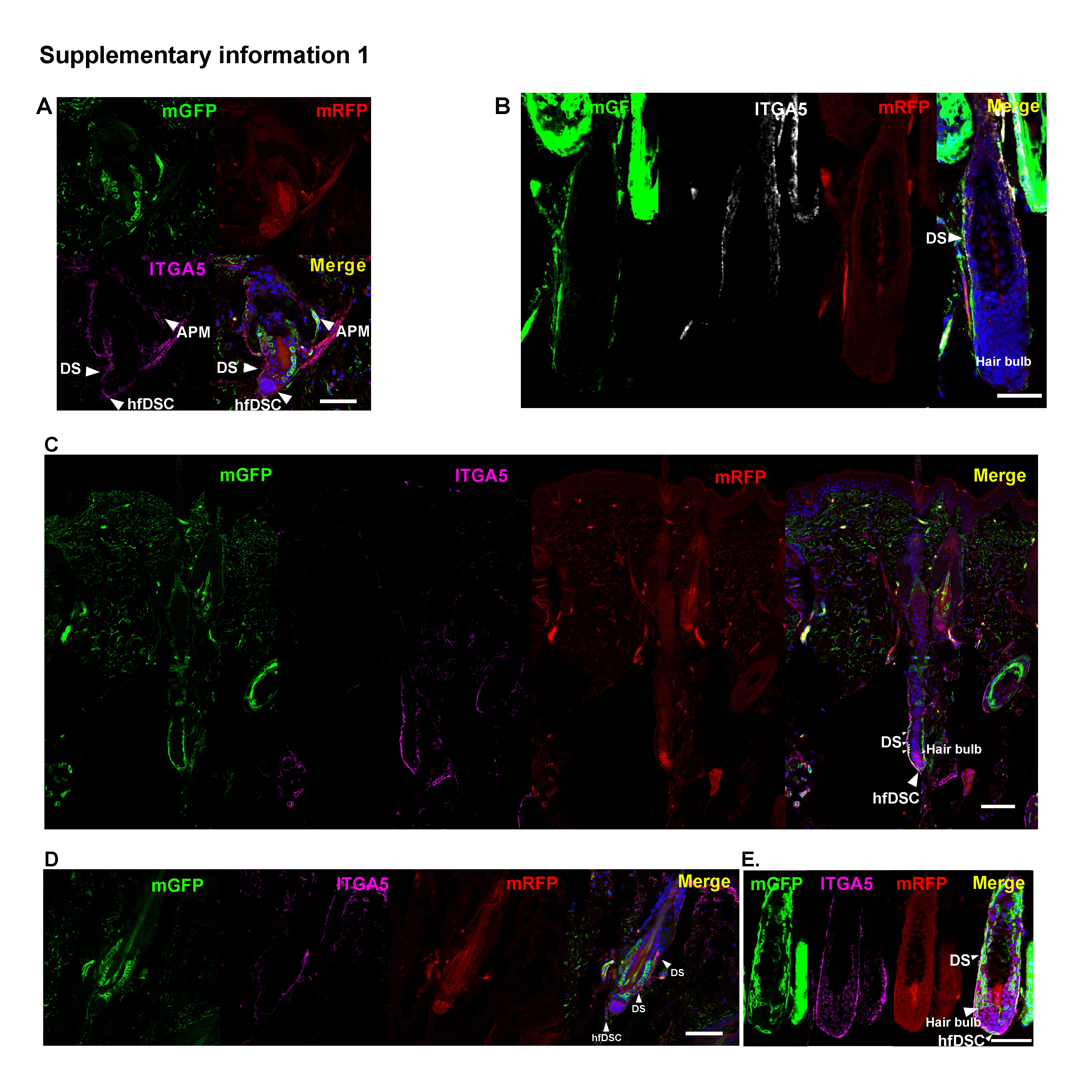

Supplement: Supplementary file 3 [file Image1.JPEG]

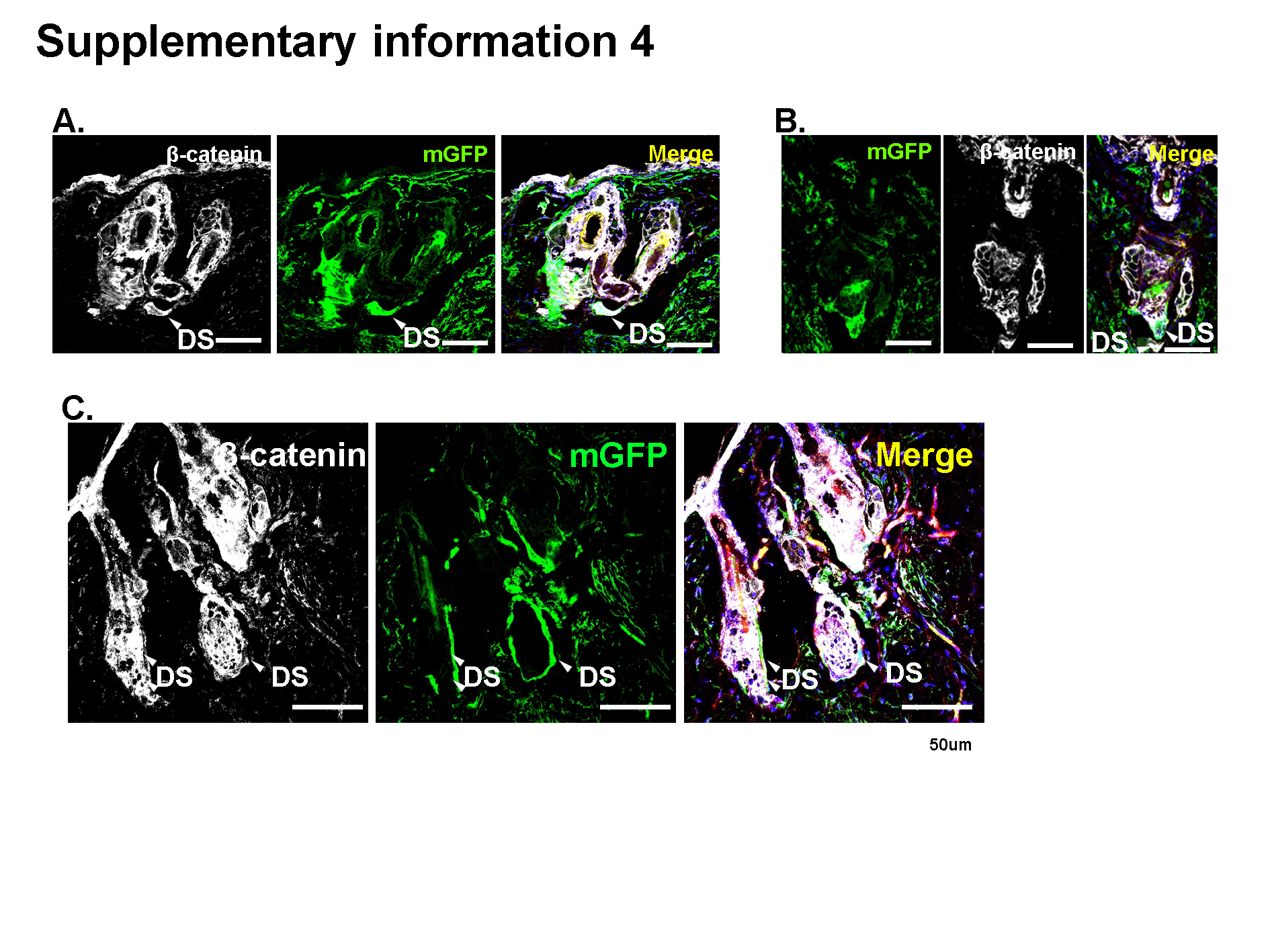

Supplement: Supplementary file 4 [file Image4.JPEG]

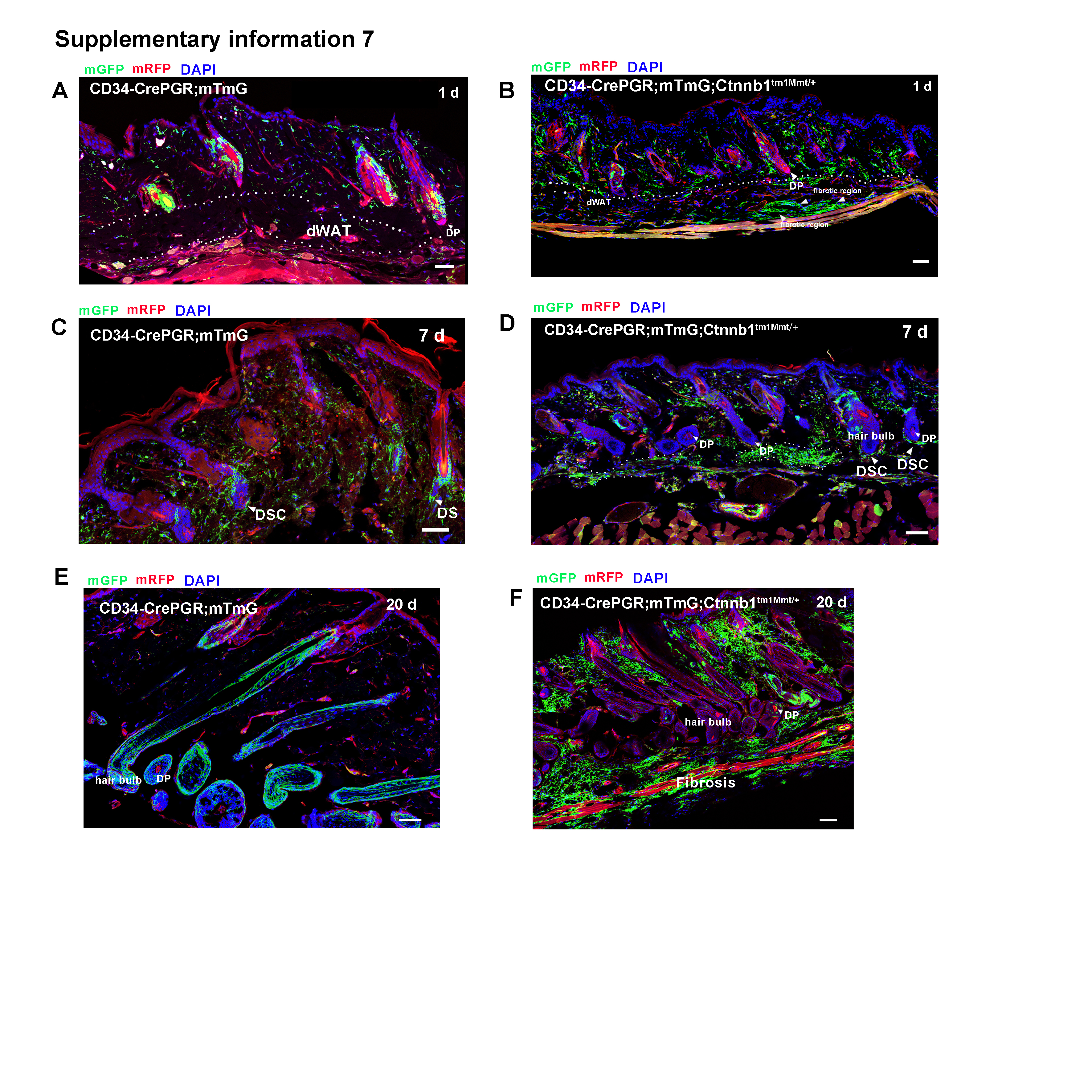

Supplement: Supplementary file 5 [file Image7.JPEG]

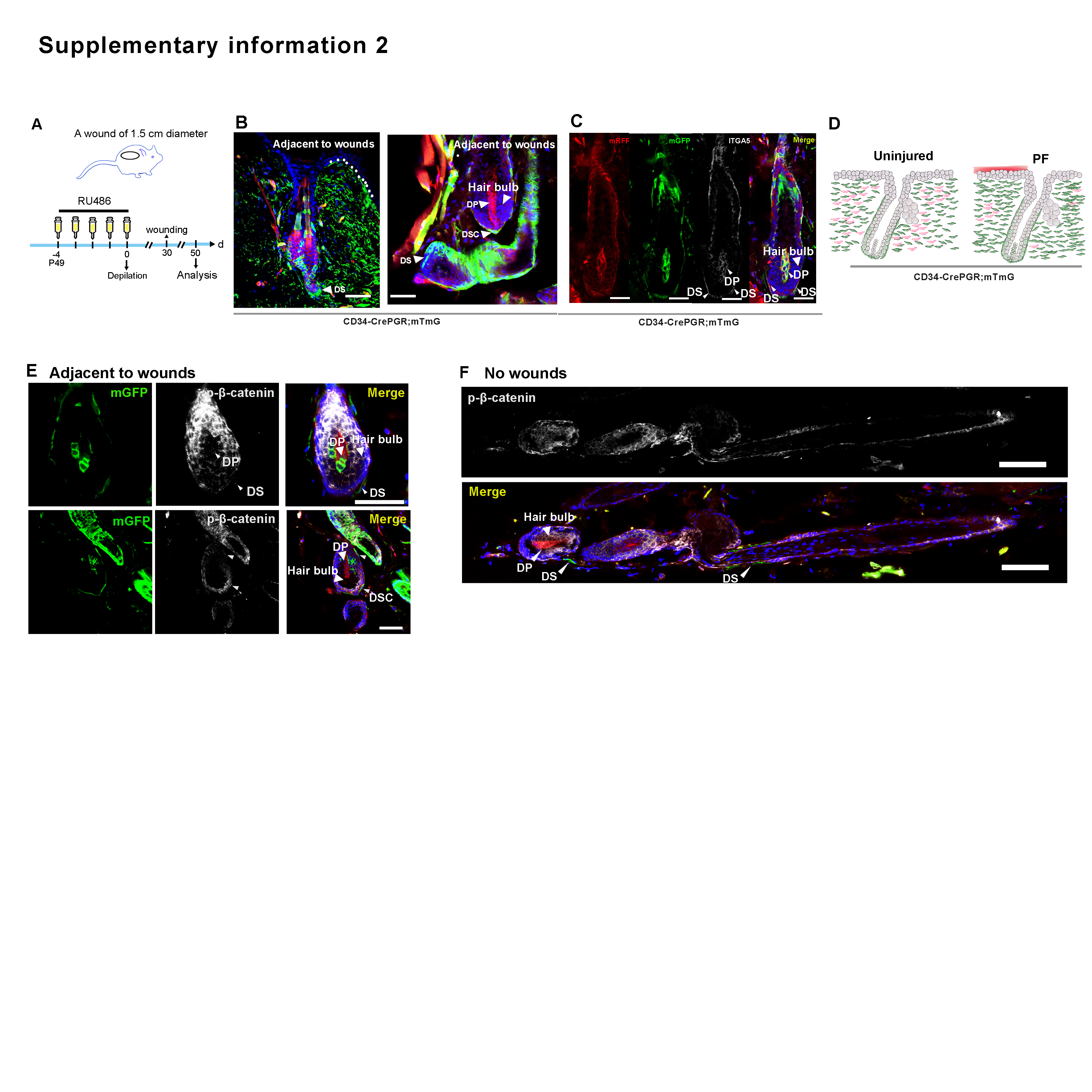

Supplement: Supplementary file 6 [file Image2.JPEG]

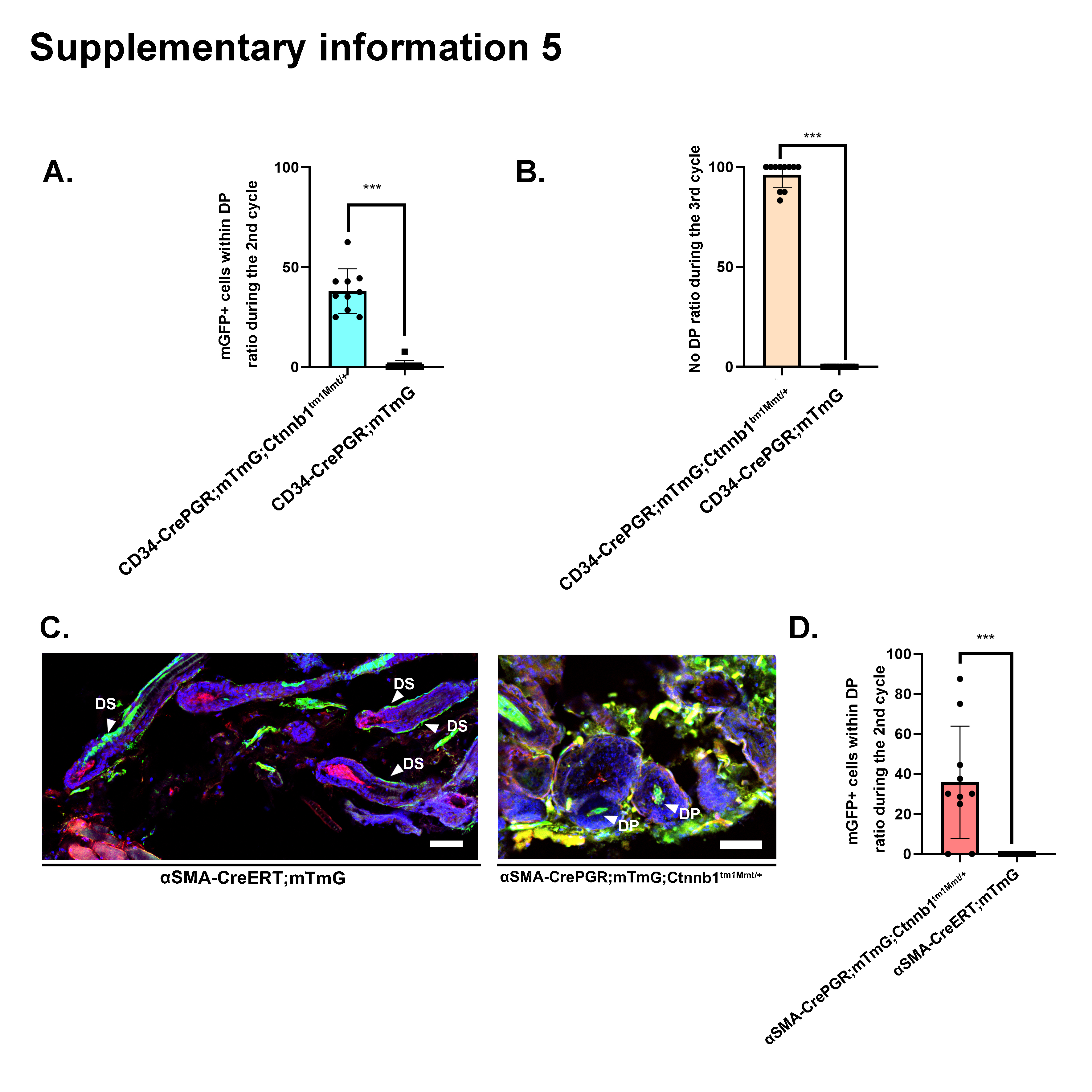

Supplement: Supplementary file 7 [file Image5.JPEG]

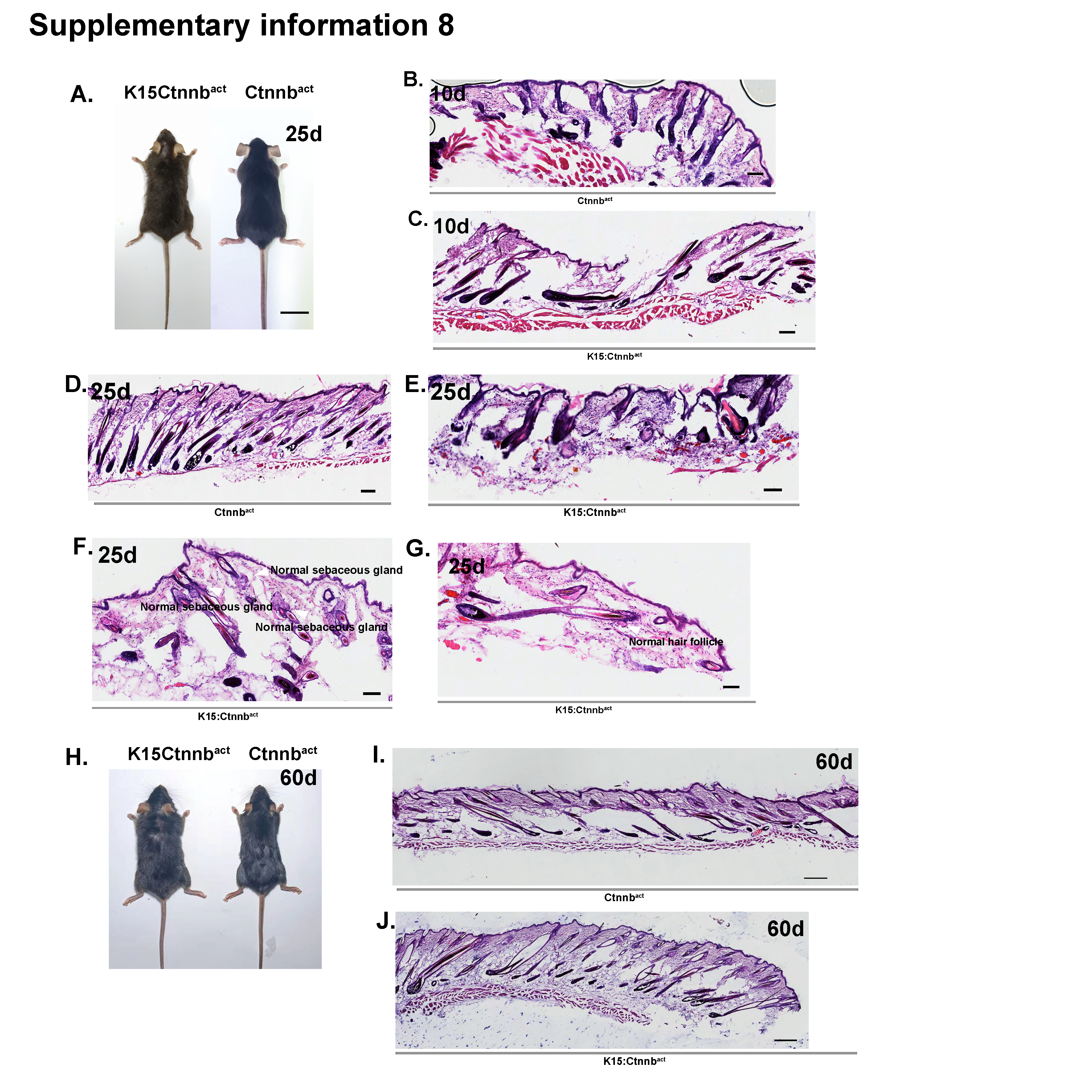

Supplement: Supplementary file 8 [file Image8.JPEG]

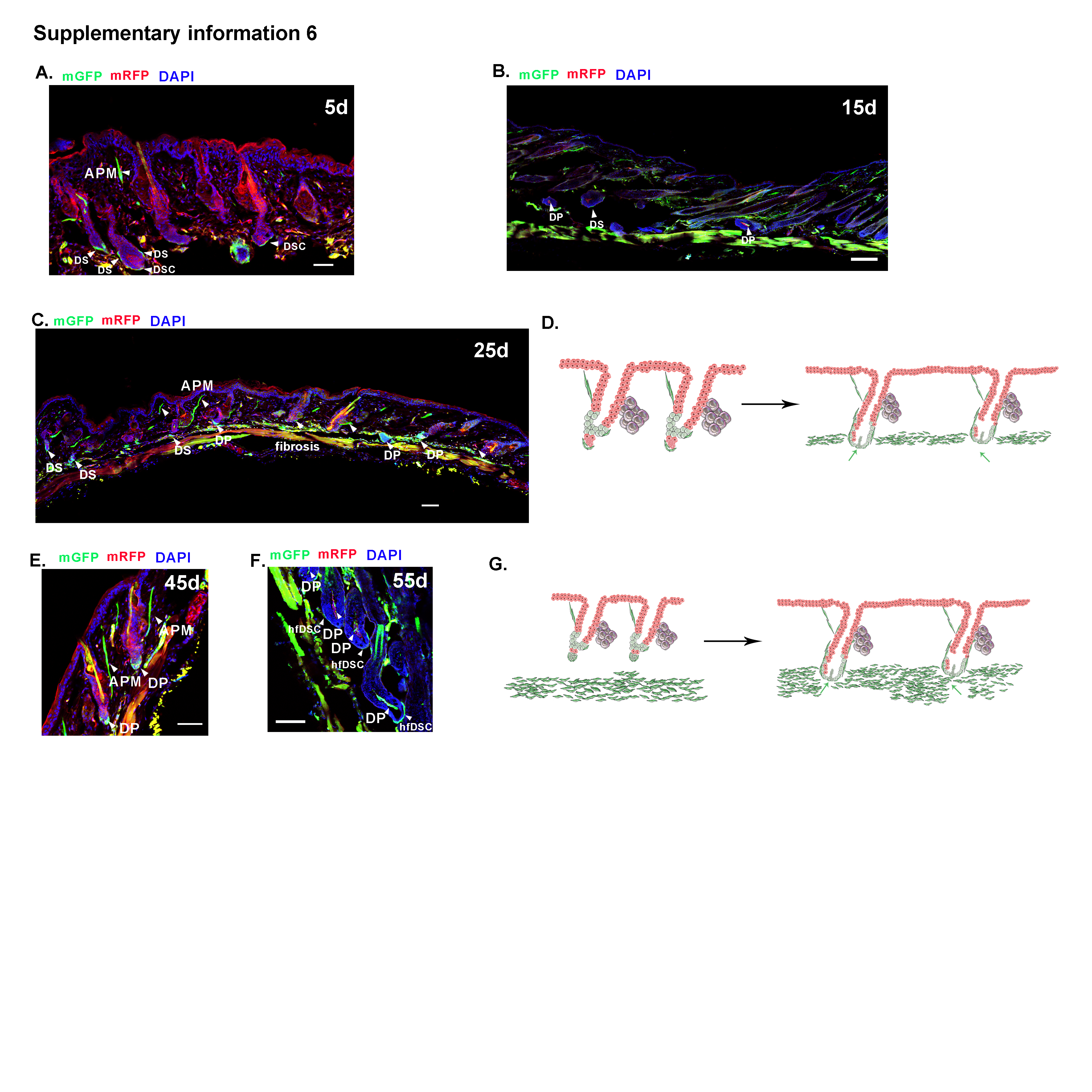

Supplement: Supplementary file 9 [file Image6.JPEG]
